# Controlled packing and single-droplet resolution of 3D-printed functional synthetic tissues

**DOI:** 10.1038/s41467-020-15953-y

**Published:** 2020-04-30

**Authors:** Alessandro Alcinesio, Oliver J. Meacock, Rebecca G. Allan, Carina Monico, Vanessa Restrepo Schild, Idil Cazimoglu, Matthew T. Cornall, Ravinash Krishna Kumar, Hagan Bayley

**Affiliations:** 1 Department of Chemistry, University of Oxford, Chemistry Research Laboratory, 12 Mansfield Road, Oxford, OX1 3TA UK; 2 Department of Zoology, University of Oxford, Zoology Research & Administration Building, 11a Mansfield Road, Oxford, OX1 3SZ UK; 3 Micron Advanced Bioimaging Unit, Department of Biochemistry, University of Oxford, South Parks Road, Oxford, OX1 3QU UK; 4 Present Address: Department of Physics and Astronomy, University of Sheffield, Sheffield, S3 7RH UK; 5 Present Address: Chemical and Physical Sciences, University of Toronto Mississauga, 3359 Mississauga Rd, Mississauga, ON L5L 1C6 Canada

**Keywords:** Membrane biophysics, Bioinspired materials, Biomimetics, Nanopores, Tissues

## Abstract

3D-printing networks of droplets connected by interface bilayers are a powerful platform to build synthetic tissues in which functionality relies on precisely ordered structures. However, the structural precision and consistency in assembling these structures is currently limited, which restricts intricate designs and the complexity of functions performed by synthetic tissues. Here, we report that the equilibrium contact angle (*θ*_DIB_) between a pair of droplets is a key parameter that dictates the tessellation and precise positioning of hundreds of picolitre-sized droplets within 3D-printed, multi-layer networks. When *θ*_DIB_ approximates the geometrically-derived critical angle (*θ*_c_) of 35.3°, the resulting networks of droplets arrange in regular hexagonal close-packed (hcp) lattices with the least fraction of defects. With this improved control over droplet packing, we can 3D-print functional synthetic tissues with single-droplet-wide conductive pathways. Our new insights into 3D droplet packing permit the fabrication of complex synthetic tissues, where precisely positioned compartments perform coordinated tasks.

## Abstract (web summary)

Precise patterning of lipid-stabilised aqueous droplets is a key challenge in building synthetic tissue designs. Here, the authors show how the interactions between pairs of droplets direct the packing of droplets within 3D-printed networks, enabling the formation of synthetic tissues with high-resolution features.

## Introduction

The packing of space-filling polyhedra in three dimensions (3D) is a common theme in nature, from the atomic packing of molecules in crystal structures^1^, to the regular arrays of cells in tissues^2^, to the macroscopic tessellation of honeycombs created by bees^3^. In these examples, distinctive properties and precise functions arise from controlled arrangement and patterning of their subunits. Similarly, the controlled close packing of deformable spheres is critical for fabricating synthetic tissues that are designed to mimic the complex and cooperative structures and functions of living tissues. In this area, networks of picolitre-sized droplets separated by droplet interface bilayers (DIBs) hold significant promise because of discrete compartmentalisation, inherent connectivity and communication between subunits^4–6^.

A DIB forms when two aqueous droplets in oil, each stabilised by a monolayer of lipids, are brought together and form a bilayer at the droplet–droplet interface (see Supplementary Note 1)^7,8^. Networks of droplets can be made based on this interfacial interaction^9^. Patterned networks of droplets have been produced by mechanical placement^9^, microfluidics^10^, optical tweezers^11^ and magnetism^12^. The most powerful approach for fabricating extended patterned networks has been droplet-based 3D-printing^4^, which provides 3D design capabilities, automation and scalability.

When two aqueous droplets come into contact and form a DIB, they deform from their initial spherical shape to form a flat bilayer interface between them. At equilibrium, the area of the lipid bilayer is reflected by a contact angle (*θ*_DIB_) measured at the point where the spherical surfaces of the two droplets meet at the flat lipid bilayer interface. When aqueous droplets pack in 3D, each makes multiple droplet–droplet contacts (by forming DIBs with its neighbours), resulting in the deformation of the spherical droplets into polyhedra. Therefore, when building a 3D network, it is crucial to understand the parameters that direct packing and droplet deformation, and thereby affect printing resolution and fidelity. At present, for example, the occurrence of printing defects dictates that conductive signalling pathways in large networks (>100 droplets) must be designed to be more than 2–3 droplets wide to ensure continuity^4,5,13^. However, single-droplet-wide signalling pathways would be feasible if synthetic tissues could be patterned at single-droplet resolution.

Various studies have aimed to understand the close packing and subsequent deformation of adhesive droplets in highly concentrated oil-in-water emulsions^14,15^. Recently, microfluidic systems have been developed to investigate how droplets pack in extended 2D sheets^16^ and how small clusters of adhesive droplets self-organise under flow^17^. Most relevant to our printed system—where droplets are placed in specific arrangements—are studies by Princen et al. on how the value of the contact angle between pairs of adhesive oil-in-water droplets controls droplet packing in small 2D and 3D assemblies^18–20^. Based on Princen’s observations and early work on adhesive water-in-oil emulsions^21,22^, we hypothesised that the value of *θ*_DIB_ between pairs of droplets would be a major geometrical constraint on the packing of hundreds of deformable aqueous droplets interfaced by lipid bilayers (see Supplementary Note 3 for discussion on geometrical constraints in droplet networks). For DIBs, *θ*_DIB_ reflects the balance of the surface tensions between the lipid monolayers and the lipid bilayer (see Supplementary Note 1 for discussion on the thermodynamics of DIB formation)^23,24^. Therefore, factors that alter the surface tensions (such as the compositions of the aqueous, lipid and oil phases), change *θ*_DIB_. Furthermore, we hypothesised that at an optimal *θ*_DIB_, droplets will regularly arrange in a hexagonal close-packed lattice (one of the optimal, ordered arrangements for close packing of spheres^25^) and space in a network would be completely filled by tessellated droplets assuming regular polyhedral geometry. Here, we demonstrate experimentally how, by controlling *θ*_DIB_, we can create regular hexagonal close-packed (hcp) lattices from hundreds of 3D-printed picolitre-sized droplets. Furthermore, we apply regular and controlled droplet packing to fabricate synthetic tissues with single-droplet-wide features in 3D, using an automated and readily scalable technology.

## Results

### *θ*_DIB_ depends on lipid and oil compositions

**Fig. 1:**
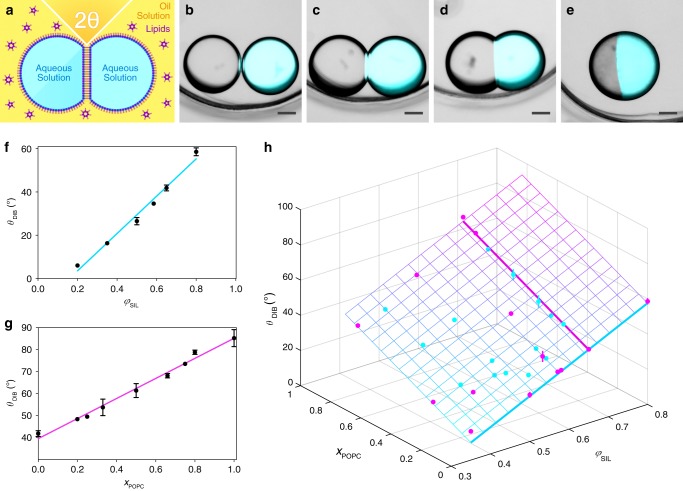
The contact angle depends on the lipid and oil compositions. **a** Schematic of a pair of aqueous droplets forming a droplet interface bilayer (DIB) in a lipid-in-oil solution, and the definition of the equilibrium contact angle (*θ*_DIB_). **b**–**e** Bright-field and fluorescence microscopy overlays of droplet pairs formed with 1 mM DPhPC and *φ*_SIL_ values of 0.20 (*θ*_DIB_ = 6.0 ± 0.7°) (**b**), 0.50 (*θ*_DIB_ = 26.5 ± 1.7°) (**c**), 0.80 (*θ*_DIB_ = 53.4 ± 0.8°) (**d**) and with 1 mM POPC and *φ*_SIL_ = 0.65 (*θ*_DIB_ = 85.3 ± 3.8°) (**e**). Scale bars = 150 µm. In each image, the right droplet contains 10 µM Atto488 to demonstrate that a bilayer has formed and compartmentalises the bilayer-impermeant dye. **f** A plot showing the linear dependence of *θ*_DIB_ with respect to *φ*_SIL_ for 1 mM DPhPC (linear regression *R*^2^ = 0.99) (see Supplementary Table 1). **g** A plot showing the linear dependence of *θ*_DIB_ with respect to *x*_POPC_ at *φ*_SIL_ = 0.65 (linear regression *R*^2^ = 0.99) (see Supplementary Table 2). **h** A plot of the 2D linear dependence of *θ*_DIB_ with respect to both *φ*_SIL_ and *x*_POPC_ (regression plane *R*^2^ = 0.99) (see Supplementary Table 8). The total lipid concentration was 1 mM. Data points that lie above and below the regression plane are in magenta and cyan, respectively. For all experiments, the aqueous phase was PBS at pH 7.2. Each data point in **f**–**h** is the mean of *n* > 3 contact angle measurements, and the error bars represent the standard deviation. When error bars are not visible, they have standard deviations smaller than the data symbols. For individual *n* values, see Supplementary Table 24.

To determine how *θ*_DIB_ depends on lipid^26^ and oil compositions^27,28^, we measured the contact angle (Fig. 1a) of pairs of 75 nL droplets containing phosphate-buffered saline (PBS, pH 7.2) under various conditions. At a total lipid concentration of 1 mM, *θ*_DIB_ depended upon both the volume fraction of silicone oil in a mixture of undecane and silicone oil (*φ*_SIL_) and the lipid composition (*x*_POPC_, the mole fraction of 1-palmitoyl-2-oleoyl-*sn*-glycero-3-phosphocholine (POPC) in a mixture of 1,2-diphytanoyl-*sn*-glycero-3-phosphocholine (DPhPC) and POPC). We found that *θ*_DIB_ is directly proportional to both *φ*_SIL_ (at 1 mM DPhPC) (Fig. 1b–d, f) and *x*_POPC_ (at *φ*_SIL_ = 0.65) (Fig. 1g), and that the maximum value of *θ*_DIB_ of 90° was approached at *φ*_SIL_ = 0.65 and *x*_POPC_ = 1.00 (Fig. 1e). Temperature (4–60 °C), droplet volume (0.52–200 nL), and total lipid concentration (1–4 mM) had no significant effect on *θ*_DIB_ (Supplementary Fig. 1, see Supplementary Note 1 for further discussion).

We used *θ*_DIB_ values from droplet pairs formed over a range of lipid and oil compositions to obtain a planar regression that accurately describes the 2D linear relationship of *θ*_DIB_ with respect to *x*_POPC_ and *φ*_SIL_ (Fig. 1h):

1 $$\theta _{\mathrm{DIB}} = \frac{{0.930\varphi _{\mathrm{SIL}} + 0.368x_{\mathrm{POPC}} - 0.238}}{{0.009}}$$

Eq. (1) allowed us to predict *θ*_DIB_ from the *φ*_SIL_ and *x*_POPC_ values used for 3D-printing droplet networks.

### 3D-printed networks contain regular and irregular packing

**Fig. 2:**
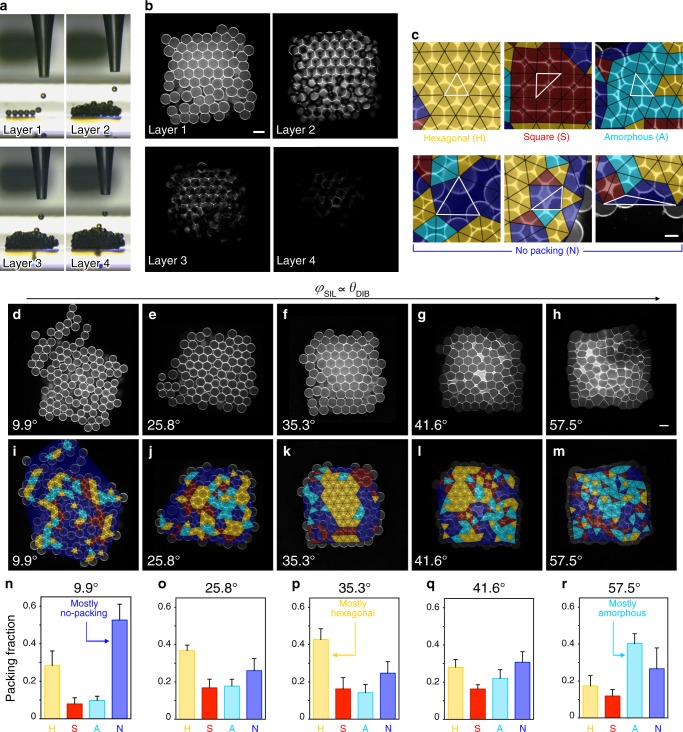
*θ*_DIB_ affects the packing of droplets in 3D-printed networks. **a** Images of a 3D-printed droplet network (7 × 8 × 4 droplets in *x, y* and *z* directions) as each layer (1 to 4) is formed. **b** Horizontal cross-sections of layer 1 (bottom), layer 2, layer 3 and layer 4 of a droplet network imaged by confocal microscopy. Lipid bilayers and monolayers are visualised with Atto550M. **c** Examples of packing types in the first layer of 3D-printed networks. Our classification method draws triangles (indicated by black and highlighted white triangles) between the centres of three neighbouring droplets and assigns the local packing type of each triplet based on the triangle geometry (Delaunay triangulation). Droplet triplets are classified as packed in a hexagonal (yellow), square (red) or amorphous (cyan) fashion, or not packed (blue) (see “Methods”). **d**–**m** Confocal microscopy images (**d**–**h**) and overlays of the packing analysis based on Delaunay triangulation (**i**–**m**) of the first layer of 3D-printed droplet networks at increasing *φ*_SIL_ values (corresponding to increasing *θ*_DIB_). Yellow, red, cyan and blue triangles represent the packing types defined as hexagonal, square, amorphous and no packing, respectively. Scale bar = 100 µm. **n**–**r** Quantification of hexagonal (yellow), square (red), amorphous (cyan) and no-packing (blue) area fractions in the first layer of 3D-printed droplet networks at increasing values of *φ*_SIL_. In **n**–**r**, each bar is the mean of *n* > 3 networks (see Supplementary Table 21 for individual *n* values and Supplementary Fig. 3); error bars represent the standard deviation.

To explore the relationship between *θ*_DIB_ and the packing arrangements of droplets, we constructed 3D droplet networks comprising hundreds of picolitre-sized aqueous droplets (PBS, 100 µm diameter, ≈524 pL volume) using our laboratory-built 3D printer^4^. To form a network, 224 droplets were automatically generated at a droplet ejection frequency of 0.5 s^−1^, and positioned line-by-line and layer-by-layer according to an hcp design (*x*, *y* and *z* dimensions of 7, 8 and 4 droplets, respectively) (Fig. 2a; Supplementary Note 2). The droplet networks were formed on plasma-treated quartz, unless otherwise stated.

To quantify packing arrangements, we imaged the interconnected phospholipid bilayers and monolayers by confocal microscopy (Fig. 2b; Supplementary Fig. 2) and assigned packing types based on Delaunay triangulation (Fig. 2c; Supplementary Fig. 3, see “Methods”)^29^. Horizontal cross-sections of the first (bottom), second, third and fourth (top) layers in droplet networks showed the 2D droplet packing within the plane of each layer (Fig. 2b). Layers two, three and four were difficult to resolve because of insufficient laser penetration and aberrations caused by the water–oil interfaces in the underlying layers (Fig. 2b; see Supplementary Note 4 for further discussions). Therefore, we quantitatively assigned packing types (see below) to areas of the first layer’s 2D packing structure. We later discuss how the packing arrangements of the first layer dictate the packing of droplets in the upper layers.

By analysing 129 printed networks (Supplementary Fig. 3), we found two prevalent regular packing arrangements that we classified as ‘hexagonal’ and ‘square’ (Fig. 2c). These correspond to ‘hexagonal close-packed’ (hcp) and ‘body-centre cubic’ (bcc) 3D lattices, respectively. We also found irregular packing arrangements, which we classified as ‘amorphous’ or ‘not packed’ depending on whether the neighbouring droplets were in contact or not (Fig. 2c).

In addition to packing arrangements, we quantified three types of defects in the first layer: fraction of the total area occupied by oil inclusions (i.e., pockets of oil trapped among surrounding droplets, Supplementary Fig. 3b, i), number of droplets that had fallen from upper layers to the bottom layer during printing (Supplementary Figs. 2a–c and 3j), and variation in droplet size (Supplementary Fig. 3k) (see “Methods” for a detailed explanation of the packing analysis).

### *θ*_DIB_ controls droplet packing in 3D-printed networks

We first determined how the packing of 3D-printed droplet networks depends on *θ*_DIB_ (calculated from Eq. (1)). When varying *φ*_SIL_ at constant lipid composition (1 mM DPhPC), we printed droplet networks at *θ*_DIB_ values that ranged from 9.9° (*φ*_SIL_ = 0.35) to 57.5° (*φ*_SIL_ = 0.8) (Fig. 2d–h).

At low *φ*_SIL_ (0.35, i.e., low *θ*_DIB_), the predominant arrangement type in droplet networks was no-packing (0.53 ± 0.08 area fraction) (Fig. 2d, i, n). Networks printed under this condition also showed abundant droplet rolling from the upper layers (65 ± 18% droplet excess) (see Supplementary Fig. 3j) and a large fraction of oil inclusions (0.18 ± 0.01 area fraction) (Supplementary Fig. 3i). In contrast, at high *φ*_SIL_ (0.80, i.e., high *θ*_DIB_), we observed abundant amorphous packing (0.40 ± 0.05 area fraction) (Fig. 2h, m, r) and droplet excess (61 ± 24%) (Supplementary Fig. 3j), together with a small fraction of oil inclusions (0.03 ± 0.01 area fraction) (Supplementary Fig. 3i).

Interestingly, at *φ*_SIL_ = 0.59 (*θ*_DIB_ = 35.3°), we found the largest fraction of hexagonal packing (0.43 ± 0.06 area fraction, Fig. 2f, k, p), together with low extents of oil inclusions (0.06 ± 0.01 area fraction) (Supplementary Fig. 3i), droplet rolling (13 ± 6% droplet excess) (Supplementary Fig. 3j), and droplet size variation (8.2 ± 1.5 coefficient of variation) (Supplementary Fig. 3k). This *θ*_DIB_ of 35.3°, equal to approximately half the dihedral angle of a regular tetrahedron (70.5°, 3 s.f.), corresponds to the geometrically calculated critical contact angle (*θ*_c_ = 35.3°, 3 s.f.) required to exclude the continuous oil phase enclosed by four droplets, when their centres are positioned at the vertices of a regular tetrahedron^20^ (see Supplementary Note 3 for derivations and Supplementary Fig. 4 for experimental evidence). When *φ*_SIL_ was increased only slightly to 0.60 (*θ*_DIB_ = 36.3°), we observed a significant drop in the hexagonal packing area fraction to 0.31 ± 0.05 (Supplementary Fig. 3e, see Supplementary Fig. 5a for accuracy of measurements).

We find that hcp is maximised in 3D-printed droplet networks when *θ*_DIB_ ≈ *θ*_c_ because, at this contact angle, the tetrahedral arrangements of droplets^30^ are maintained in the position imposed by the printing nozzle with minimal distortions. In a pair of droplets forming a DIB, the adhesive energy of the system (*ΔF*, defined as the work required to form a lipid bilayer per unit area) increases with increasing bilayer area, and therefore with increasing *θ*_DIB_ (Supplementary Fig. 1f, Supplementary Eq. 3). Consequently, larger values of *θ*_DIB_ in 3D-printed networks lead to increasingly cohesive and compact droplet assemblies. However, when a DIB is formed, the two droplets reduce their centre-to-centre distance as a consequence of the deformation of the droplets at the bilayer interface (Fig. 1a). The centre-to-centre distance decreases with increasing *θ*_DIB_ (Supplementary Eq. 4). When *θ*_DIB_ >> *θ*_c_, large reductions in centre-to-centre distances affect the precise positioning and packing of droplets in networks, resulting in irregular arrangements (Fig. 2h, m, r). Conversely, when *θ*_DIB_ << *θ*_c_, the adhesive energy of the system is too low to form self-supporting 3D structures (Fig. 2d, i, n; Supplementary Fig. 4), resulting in distorted and loosely packed networks. For *θ*_DIB_ ≈ *θ*_c_, hcp is maximised in 3D-printed droplet networks because the adhesive energy between droplets is sufficient to allow formation of self-supporting 3D structures, and droplets are only minimally displaced from their initial positions (imposed by the printing nozzle) when they attain their final positions, with centre-to-centre droplet distances optimal for hcp (once *θ*_DIB_ is reached) (Fig. 2f, k, p, see Supplementary Note 3 for a detailed mechanistic and geometrical explanation).

Taken together, these results (for 1 mM DPhPC and various *φ*_SIL_ values) reveal three *θ*_DIB_-dependent situations: *θ*_DIB_ << *θ*_c_, droplet networks pack loosely with the greatest amount of no packing (Fig. 2n); *θ*_DIB_ >> *θ*_c_, droplet networks pack tightly and are distorted, with the greatest amount of amorphous packing (Fig. 2r); and *θ*_DIB_ ≈ *θ*_c_, droplet networks show the greatest amount of hexagonal packing (Fig. 2p).

### The kinetics of DIB formation affect hexagonal packing

**Fig. 3:**
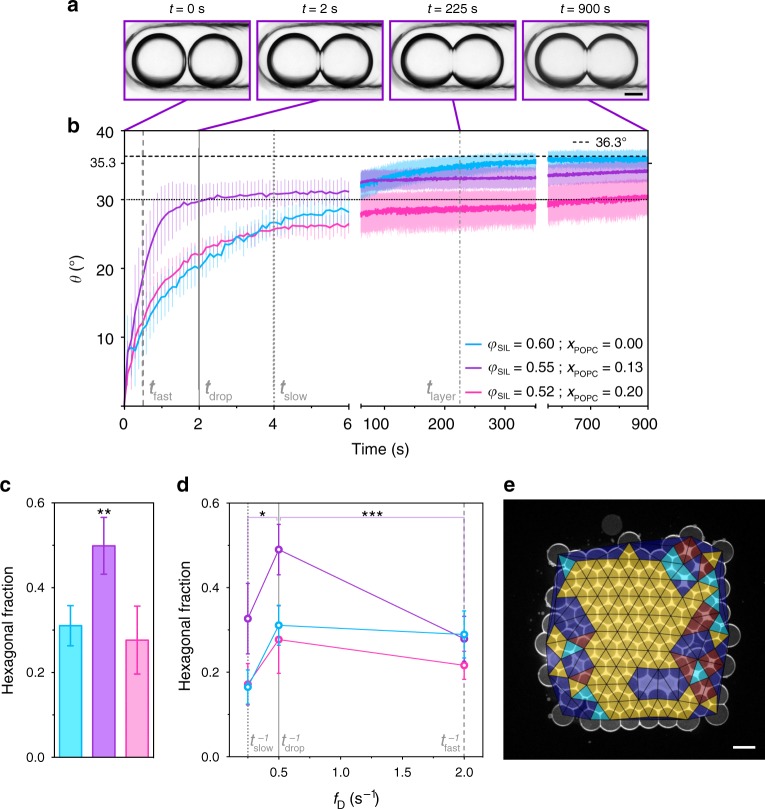
The kinetics of contact angle equilibration affects hexagonal packing. For all conditions in **a**–**e**, *θ*_DIB_ = 36.3° (calculated from Eq. (1)). The *φ*_SIL_ and *x*_POPC_ values in **b**–**d** are *φ*_SIL_ = 0.60 and *x*_POPC_ = 0.00 (cyan), *φ*_SIL_ = 0.55 and *x*_POPC_ = 0.13 (purple), and *φ*_SIL_ = 0.52 and *x*_POPC_ = 0.20 (magenta). **a** Optical microscopy images of two droplets (75 nL) forming a DIB after contact. The non-equilibrium contact angle (*θ*) increases with time (0–900 s) until the equilibrium contact angle (*θ*_DIB_) is reached (*t* = 1800 s). Scale bar = 150 µm. The images correspond to timepoints along the purple profile in **b**. **b** Plots of *θ* versus time for droplet pairs. Plots are the mean of *n* = 5 repeats for each condition, and error bars represent the standard deviation. The grey dashed lines correspond to timepoints relevant to printing 3D droplet networks: *t*_fast_ (0.50 s), *t*_drop_ (2.00 s) and *t*_slow_ (4.00 s) indicate the time intervals between consecutive printed droplets at fast (*t*_fast_^−1^ = 2.00 s^−1^), standard (*t*_drop_^−1^ = 0.50 s^−1^), and slow (*t*_slow_^−1^ = 0.25 s^−1^) printing frequencies; *t*_layer_ (225 s) is the time it takes to print a single layer at a printing frequency of 0.50 s^−1^. **c** A bar chart of the hexagonal packing area fraction of 3D droplet networks printed at a droplet ejection frequency of 0.50 s^−1^. The hexagonal area fraction under the purple condition was significantly greater than for the other two conditions (one-way ANOVA with Tukey’s multiple comparison test) (see Supplementary Table 15). **d** Plots of hexagonal packing area fractions in the first layers of 3D droplet networks generated at different printing frequencies (*f*_D_). The frequencies marked in grey are the inverse of the timepoints marked in **b** (i.e., *f*_D_ = *t*_D_^−1^, where *t*_D_ is the time interval between the ejection of two consecutive droplets). For statistical tests, see Supplementary Table 16. **e** A confocal microscopy image and overlay of Delaunay triangulation of the first layer in a 3D-printed droplet network (7 × 8 × 4; *x*, *y*, *z*) at *φ*_SIL_ =  0.55 and *x*_POPC_ = 0.13 (purple condition in **b**–**d**) at a printing frequency of 0.50 s^−1^. Scale bar = 100 µm. Each data point in the graphs **c** and **d** is the mean of *n* > 3 repeats, and error bars represent the standard deviation. *, ** and *** indicate *p*-value <0.05, 0.01 and 0.001, respectively. For individual *n* values, see Supplementary Tables 22 and 23.

Since the finding that *θ*_DIB_ should approximate *θ*_c_ was critical for maximising hexagonal packing in the first layer, we hypothesised that the packing of droplets in 3D-printed networks would also depend on the kinetics of DIB formation, i.e. the time taken for a pair of droplets to reach *θ*_DIB_ after contact. We therefore investigated the impact of the lipid and oil compositions on the kinetics of DIB formation by monitoring the changes in the non-equilibrium contact angle (*θ*) over time between two droplets under various conditions (Fig. 3; Supplementary Note 3 and Supplementary Fig. 6). Importantly, the same *θ*_DIB_ value of 36.3° was used in each experiment by adjusting the ratio of *x*_POPC_ to *φ*_SIL_ (Fig. 1h).

When POPC was included in the lipid mixture, the rate of contact angle equilibration was biphasic—an initial faster phase was followed by a slower phase (Fig. 3a, b). For example, at *φ*_SIL_ = 0.55 and *x*_POPC_ = 0.13, droplet pairs reached a non-equilibrium contact angle of 30° within 2.00 s after contact, but then took over 15 min to reach a *θ*_DIB_ value of 36.3° (Fig. 3a, b). When we generated droplet networks under these conditions, with a printing frequency that matched the time taken to reach the non-equilibrium contact angle of 30° (*t*_drop_ = 2.00 s, corresponding to a printing frequency *t*_drop_^−1^ = 0.50 s^−1^), we obtained the largest fraction of hexagonal packing (0.50 ± 0.07 area fraction) in the networks (Fig. 3c–e). Specifically, by the time a new droplet was ejected (*t*_drop_ in Fig. 3b), the contact angle at the previous droplet–droplet interface had reached a non-equilibrium value of 30°. Since this value of 30° is optimal for 2D hexagonal packing of droplets (Supplementary Note 3), the matching of the printing frequency and the fast phase of DIB formation allowed the first layer to pack hexagonally before the second layer was printed (Fig. 3b, the time taken to print a layer was *t*_layer_ = 225 s). The slow phase in DIB formation allowed the slow development of hcp from the hexagonal packing in the first layer, as the non-equilibrium contact angle reached its final equilibrium value of 36.3° (≈ *θ*_c_). We further confirmed this by observing that deviations from a printing frequency of 0.50 s^−1^ significantly reduced hexagonal packing in the networks printed at the same lipid–oil composition (Fig. 3d).

These experiments confirmed our hypotheses that the regularity of the hcp in 3D-printed droplet networks was optimal when *θ*_DIB_ ≈ *θ*_c_, and also when the printing frequency and the kinetics of DIB formation were matched to allow the initial formation of regular 2D hexagonal packing in the first layer, which then templated hcp when subsequent layers were printed on top.

### Packing arrangements localise in specific regions

**Fig. 4:**
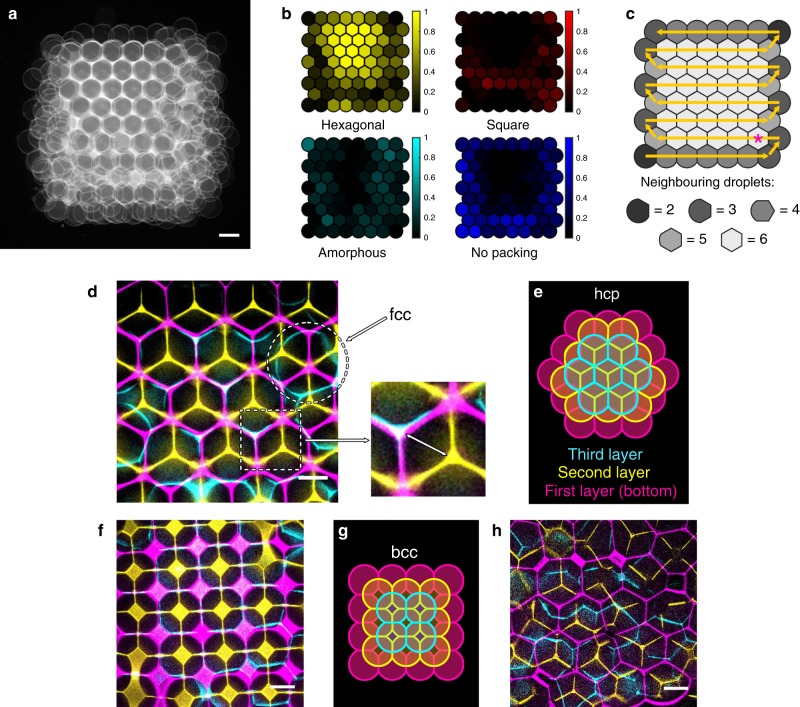
Packing arrangements in the first layer propagate to the upper layers. **a** Overlaid confocal microscopy images (co-registered) (*n* = 5) of the first layer of 3D-printed droplet networks, which had a hexagonal packing fraction of 0.50 ± 0.07 (*θ*_DIB_ = 36.3°, *φ*_SIL_ = 0.55, *x*_POPC_ = 0.13). **b** Heatmaps of the 2D localisation of different types of packing in the first layer generated by analysing the images in **a**. The relative occurrence of each packing type (yellow, red, cyan and blue represent hexagonal, square, amorphous and no packing, respectively) is binned onto an idealised printing map of the first layer (see “Methods”). **c** A diagram of the droplet printing path in the first layer (yellow arrows), with droplet shapes corresponding to the number of neighbouring droplets. The star indicates the first droplet in the printing path to become surrounded by six neighbouring droplets. **d** Overlaid confocal microscopy images of horizontal cross-sections of the first (magenta), second (yellow) and third (cyan) layers of a single 3D-printed droplet network (*φ*_SIL_ = 0.55, *x*_POPC_ = 0.13). The zoomed-in panel shows that layers 1 and 3 are in alignment, while layer 2 is offset by a distance equal to the circumradius of the unit hexagon (marked by the white arrow). The circle marks a face-centred cubic defect. **e** A diagram of hexagonal close packing corresponding to **d**. **f** Overlaid confocal microscopy images of the first (magenta), second (yellow) and third (cyan) layers of a single 3D-printed droplet network derived from a patch of square packing in the first layer (*φ*_SIL_ = 0.50, *x*_POPC_ = 0.27). Similarly to **d**, the overlay shows that layer 2 is offset with respect to layers 1 and 3. **g**, A diagram of body-centred cubic (bcc) packing corresponding to **f**. **h** Overlaid confocal microscopy images of the first (magenta), second (yellow), and third (cyan) layers of a single 3D-printed droplet network derived from an amorphous patch in the first layer (*φ*_SIL_ = 0.65, *x*_POPC_ = 0.33). No regular lattice is formed between the layers. Scale bars are 100 µm for **a**; and 50 µm for **d**, **f**, and **h**. **d**, **f** and **h** are from three different droplet networks.

Our optimised conditions for obtaining hcp lattices increased network regularity. To study whether ordered and disordered patches localised to specific regions of printed networks (e.g., centre versus edges), we overlaid 2D cross-sectional images of the first layers of droplet networks printed at *φ*_SIL_ = 0.55 and *x*_POPC_ = 0.13 (Fig. 4a), and mapped the occurrence of different types of packing onto an idealised map of the first layer (see “Methods”) (Fig. 4b).

We observed that droplets were mostly hexagonally packed throughout the networks, while square, amorphous and no-packing arrangements were confined to the edges (Fig. 4b). Due to their positions, droplets at the edges of the networks were coordinated to less than six neighbouring droplets in the first layer (Fig. 4c), and as a result often exhibited irregular packing (amorphous and no packing) (Fig. 4b). Conversely, droplets that were coordinated to six neighbours predominantly exhibited hexagonal packing (Fig. 4b). Once a small cluster of droplets is hexagonally packed during the printing process, it may serve as a nucleation event, propagating hexagonal packing to neighbouring areas and thereby producing extended hexagonal regions (Fig. 4c).

In summary, droplets surrounded by six others predominantly formed extended hexagonally packed regions. In contrast, irregular packing was confined to the periphery of the printed networks, where droplets were surrounded by fewer than six neighbours.

### Packing arrangements propagate upwards

By using overlays of 2D cross-sections of the first, second and third layers, we observed the spatial propagation of packing arrangements from the first layer to the upper layers (Fig. 4d–h). For hexagonal lattices (Fig. 4d), droplets in layers 1 and 3 were in alignment with each other, but offset with respect to droplets in layer 2 by centre-to-centre distances equal to the circumradius of a unit hexagon (defined in Fig. 4d), in accordance with an hcp lattice (Fig. 4e). Within hexagonal regions, we sometimes found droplets in the third layer offset with respect to both layers 1 and 2, corresponding to face-centred cubic arrangements (Fig. 4d).

Non-hcp regions were also propagated from the first layer to the upper layers. For instance, sections of square packing in the first layer propagated into body-centred cubic packing arrangements (Fig. 4f, g). In disordered sections of the first layer, and generally when *θ*_DIB_ >> *θ*_c_ (amorphous packing), we found that disordered packing arrangements propagated into the upper layers (Fig. 4h).

In summary, packing arrangements in the first layer propagated into the upper layers, stressing the importance of regular packing of droplets in the first layer. The surface onto which the network was printed played an important role in supporting an ordered first layer. Droplets printed on plasma-treated quartz formed an adhesive patch (likely a lipid bilayer^31^) with the surface, which constrained droplets in position while printing. In contrast, droplets printed on non-treated quartz or rough glass formed less regular arrangements (Supplementary Fig. 7).

### 3D replicas of tessellated droplet shapes confirm hcp

**Fig. 5:**
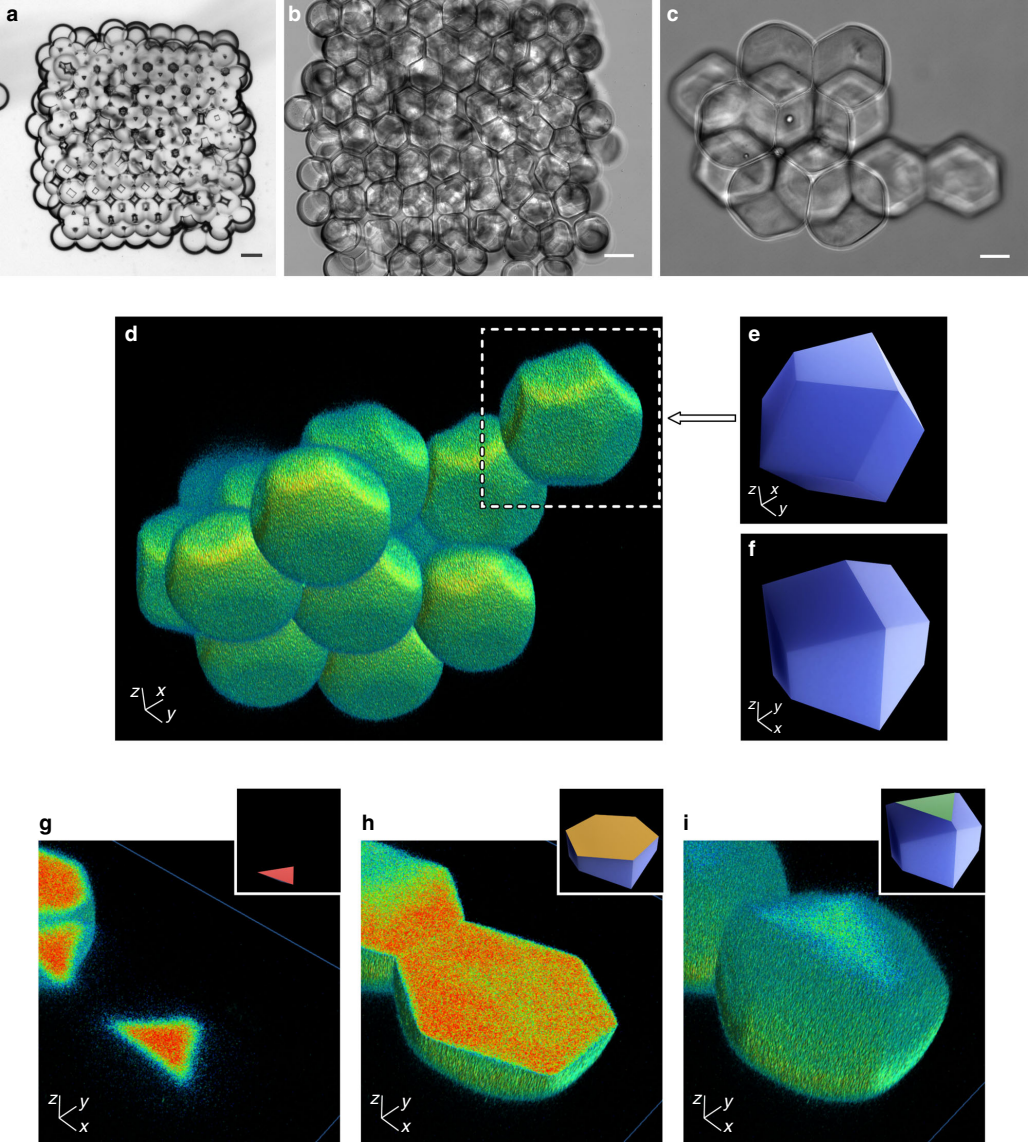
The geometry of droplet replicas confirms hexagonal close packing. **a**–**c** Bright-field microscopy images of a 3D-printed droplet network (10 × 12 × 4; *x*, *y*, *z*) (1 mM DPhPC, *φ*_SIL_ = 0.60, and a calculated *θ*_DIB_ = 36.3° from Supplementary Fig. 5b) comprising an aqueous phase of 20% (w/v) poly(ethylene glycol) diacrylate, 0.5% (w/v) Irgacure 2959 (photo-initiator), 100 µM ethidium bromide-*N,N’*-bisacrylamide (photo-cross-linkable fluorophore), and PBS, before (**a**) and after (**b**) photo-polymerisation with UV light. Scale bars = 100 µm**. c** An image of an hcp cluster of hydrogel polyhedra dispersed in PBS. Scale bar = 25 µm. **d** 3D reconstruction of droplet shapes from confocal microscopy of the hcp region in **c**, which contained 14 clustered droplets (Supplementary Movie 1). **e**, **f** A computer model of a trapezo-rhombic dodecahedron—the space-filling polyhedron of hexagonal close packing—viewed from below (**e**) (compare the white box in **d**) and above (**f**). **g**–**i** A droplet sectioned through the *z*-axis from bottom to top, with (inset) computer models of trapezo-rhombic dodecahedra showing 2D sections of three-fold (**g**, **i**) and six-fold (**h**) symmetry.

To confirm the hcp of droplets in 3D-printed networks, we imaged the space-filling shapes adopted by the droplets within a network. The final shape of a droplet is determined by *θ*_DIB_, and by the number of surrounding droplets and their locations. Optical aberrations caused by water–oil interfaces prevented us from directly imaging 3D shapes of individual fluorescent droplets within printed networks by optical microscopy (Supplementary Note 4 and Supplementary Fig. 8). Instead, we created fluorescent-poly(ethylene glycol) hydrogel replicas of droplets in networks, dispersed these replicas in PBS and reconstructed their 3D geometries from *z*-stacks acquired by confocal microscopy (Fig. 5a–c; see “Methods”). Droplet replicas were often attached to each other, thereby representing the 3D packing structure we observed in droplet networks (Fig. 5d; Supplementary Fig. 9 and Supplementary Movies 1 and 2).

In hexagonally packed regions of droplet network replicas, a droplet in the second or third layer was surrounded by 12 droplets, which formed 12 DIBs—producing the space-filling trapezo-rhombic dodecahedron (*D*_*3h*_) (Fig. 5d) expected for hcp^32^. Horizontal cross-sections of these polyhedra showed a regular hexagon at the midpoint (Fig. 5h), with two aligned equilateral triangles near the top and bottom (Fig. 5g, i). In contrast to what we saw for hcp, horizontal sections of a polyhedron in a face-centred cubic lattice—a rhombic dodecahedron (*O*_*h*_)—would show two equilateral triangles, near the top and the bottom of the polyhedron, rotated by 60° with respect to each other. Droplets at the bottom of a network (touching the glass) were surrounded by nine droplets and formed ten-faced polyhedra (*C*_*3v*_) with a flat circular face due to bilayer formation at the quartz surface (Supplementary Fig. 9h–k). Droplets at the periphery (at the top and sides of a network) formed polyhedra with ten faces or less, and with a curved face arising from the oil–water interface (Supplementary Fig. 9l–p).

### Single-droplet-wide features in synthetic tissues

Finally, we applied our findings on regular droplet packing to the fabrication of synthetic tissues with high-resolution functional features in 3D. In biological systems, complex functionalities emerge not only from the cell types present but also from the coordinated interaction of simpler functions performed by specialised cells organised in defined architectures^33^. When designing synthetic tissues, we take inspiration from biological systems to develop artificial systems with behaviour not restricted to biological or biomimetic functions^4,5,9,34^.

**Fig. 6:**
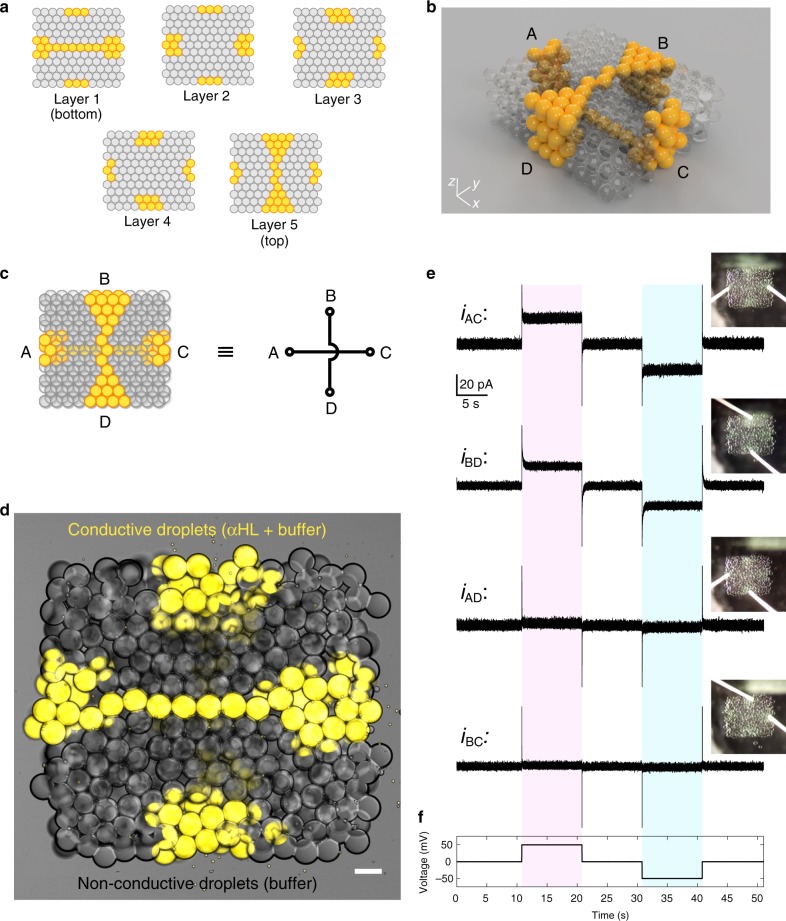
Synthetic tissues with features patterned at single-droplet resolution. **a** Maps of the first (bottom), second, third, fourth and fifth (top) layers of a 3D-printed synthetic tissue in which two conductive, single-droplet-wide pathways containing αHL (in yellow) are patterned in 3D within a network of non-conductive droplets (in grey). **b** A computer model displaying the 3D architecture of the synthetic tissue. The two single-droplet-wide pathways span the synthetic tissue, one horizontally at the bottom (connecting A to C), and one vertically at the top (connecting B to D) of the network. **c** Simplified diagram of the synthetic tissue, and equivalent circuit model. **d** Bright-field and fluorescent microscopy overlay of the synthetic tissue. Droplets in the single-droplet-wide conductive pathways contained α-hemolysin (60 µg mL^−1^), 25 mM Tris-HCl (pH 7.6), 1 M NaCl and 10 µM Atto488 fluorophore (false coloured in yellow). Scale bar = 100 µm. **e** Electrical recordings of the ionic currents flowing through the two single-droplet-wide conductive pathways connecting A to C (*i*_AC_) and B to D (*i*_BD_) upon application of the voltage protocol shown in **f**. When a potential of ± 50 mV was applied, changes in ionic currents of +25.6 ± 1.4 pA (at positive potential) and −25.6 ± 1.5 pA (at negative potential) were observed for *i*_AC_, and of +19.4 ± 1.2 pA (at positive potential) and −19.8 ± 1.3 pA (at negative potential) for *i*_BD_. Conversely, we recorded non-significant changes in ionic current for the same applied potentials between A and D (*i*_AD_) or B and C (*i*_BC_). *i*_AD_: +1.8 ± 1.3 pA and −1.8 ± 1.4 pA (at positive and negative potentials respectively); *i*_BC_: +0.9 ± 1.1 pA and −0.9 ± 1.1 pA (at positive and negative potentials, respectively).

To demonstrate how spatially controlled droplet packing defines functionality, we designed a synthetic tissue whose function directly emerged from the correct positioning of individual droplets (by applying *θ*_DIB_ ≈ *θ*_c_ and confining high-resolution patterns to the centre of the construct) (Fig. 6). Specifically, this construct featured two separate, electrically conductive, single-droplet-wide pathways (composed of droplets containing the ion-permeable membrane protein α-hemolysin^12^, αHL), which were patterned in 3D within a network of non-conductive droplets (which did not contain αHL). Based on the pore formation mechanism of αHL in lipid bilayers^12^, the two conductive pathways in our design were separated by three insulating droplet layers to electrically isolate the two pathways from each other. The two pathways spanned the construct within different droplet layers: one horizontally at the bottom of the network (within layer 1, connecting A to C in Fig. 6b, c) and one vertically at the top of the network (within layer 5, connecting B to D in Fig. 6b, c). Imperfections in the droplet lattice would lead to either interruption of the electrical signal along the single-droplet-wide conductive pathways, or crosstalk between the two pathways designed as distinct (see circuit model in Fig. 6c). Therefore, electrical recordings would give us a direct readout of the precisely controlled packing of droplets in this construct.

By confocal microscopy, we observed the designed configuration of the single-droplet-wide conductive pathway at the bottom of the construct (Fig. 6d). We could not resolve the upper conductive pathway because of the imaging limitations discussed above (Supplementary Note 4). Interestingly, we observed that αHL-containing droplets showed a smaller *θ*_DIB_ compared with droplets not containing αHL. We attributed this decrease in *θ*_DIB_ to the interaction of αHL with the lipid monolayers and bilayers, leading to changes in surface tension. However, the neighbouring buffer droplets, for which *θ*_DIB_ ≈ *θ*_c_, packed as an hcp lattice (in the centre of the construct) and constrained the αHL-containing droplets to an hcp lattice, which consequently maintained precise interconnection of the single-droplet-wide conductive pathway (Fig. 6d).

To confirm the correct functionality of the synthetic tissue, we performed electrical recordings. When a potential of ± 50 mV was applied, we detected ionic currents flowing through the bottom and top single-droplet-wide pathways, i.e., from A to C (*i*_AC_) and from B to D (*i*_BD_) (Fig. 6e). By contrast, we detected no ionic current when we applied a potential between A and D or B and C (*i*_AD_ and *i*_BC_), demonstrating that the two orthogonal pathways were spatially and functionally distinct, in accordance with our design. Taken together, these experiments demonstrate that functional synthetic tissues can be created at single-droplet resolution (100 µm diameter, ≈524 pL voxel volume) in three dimensions using our droplet printer. To our knowledge, this is the first example of an automated system generating such well-controlled droplet packing and patterning on the micrometre scale.

## Discussion

In summary, we found that 3D-printed droplet networks adopted hcp lattices when *θ*_DIB_ in droplet pairs approximated the geometrically derived critical angle (*θ*_c_) of 35.3° (Fig. 2). Deviations from this critical angle led to networks that were either loosely packed (*θ*_DIB_ << 35.3°) or tightly packed and distorted (*θ*_DIB_ >> 35.3°), both with increased lattice defects. We also noted a greater extent of hexagonal packing when the printing frequency was matched to the rapid phase of DIB formation to allow regular 2D hexagonal packing to form in the first layer before the upper layers were printed. The rapid phase of contact was followed by the slow formation of 3D hcp droplet lattices as the non-equilibrium contact angle approached *θ*_DIB_ ≈ *θ*_c_ (Fig. 3). In addition, regular hexagonal packing of the first layer was crucial because packing arrangements propagated into the upper layers (Fig. 4). In hcp regions, droplets formed space-filling trapezo-rhombic dodecahedra that featured 12 DIBs with the surrounding droplets (Fig. 5). Our findings are applicable to any other assemblies of compartmentalised systems—such as adhesive giant unilamellar vesicles^35^ or protein compartments^36^—in which structural order is required to build synthetic tissues with precise functionalities.

Based on our improved control over the packing of droplets within 3D-printed networks, we can fabricate 3D-printed droplet networks with intricate designs at high resolution (e.g., tubular structures, Supplementary Fig. 11). We exemplified this by building a synthetic tissue containing two single-droplet-wide conductive pathways with a minimal separation of three insulating droplet layers, confirming that we are able to pattern functional synthetic tissues with single-droplet-resolution features in 3D (Fig. 6). This level of precision was not achieved at the periphery of the 3D-printed constructs, where most printing defects and irregular arrangements of droplets were confined (Figs. 4, 6). However, our observations also suggest that regular packing at the periphery of 3D-printed droplet networks might be further improved by ‘annealing’ steps after the printing process (i.e., cyclical decrease and increase in contact angles), or by templating the droplet packing lattice using patterned surfaces.

Our findings show that it is possible to reproducibly pattern droplet networks with single-droplet precision by using an automated and readily scalable technology. Our work provides new knowledge about the parameters that drive droplet packing and opens up new possibilities, such as the fabrication of increasingly complex synthetic tissues that can reproduce the structurally coordinated interactions of living cells. This improved understanding will also allow the fabrication of precisely and reproducibly constructed 3D-printed structures containing eukaryotic and prokaryotic cells to study tissue development, cellular ecology and disease models^13,37^. Further, our precise patterning and packing provides a basis for accurately interfacing 3D-printed synthetic constructs with living tissues—offering new means to monitor, control or complement biological function.

## Methods

### Lipid–oil solutions

Lipids were purchased from Avanti Polar Lipids and stored as stock solutions in chloroform at a concentration of 25 mg mL^−1^ at −20 °C in 1.5 mL Teflon-capped glass vials (Supelco). Lipid stock solutions were used within a month of preparation. Undecane and silicone oil AR20 (Sigma-Aldrich) were filtered before use through 0.22 µm filters (Corning) under vacuum. Lipid–oil solutions were prepared by evaporating desired amounts of lipid in chloroform (1,2-diphytanoyl-*sn*-glycero-3-phosphocholine (DPhPC), 1,2-dioleoyl-*sn*-glycero-3-phosphocholine (DOPC), 1-palmitoyl-2-oleoyl-*sn*-glycero-3-phosphocholine (POPC) and 1,2-dimyristoyl-*sn*-glycero-3-phosphocholine (DMPC)) under a slow stream of nitrogen while rotating by hand in isopropanol-cleaned glass vials to produce an even lipid film. The film was dried under vacuum for 30 min followed by addition of oils at a desired ratio and sonication for 30 min before use. Lipid–oil solutions were used within a week.

### Aqueous solutions

Phosphate buffer saline (PBS, pH 7.2) (Sigma-Aldrich) was filtered through a 0.22 µm filter (Millex) before use. Where indicated, Atto488 and Atto550M (Sigma-Aldrich) were dissolved in PBS at 10 µM and 3 µM, respectively. Fluorescent dyes were diluted in PBS from stock solutions in DMSO (Sigma-Aldrich). Stock solutions were prepared at concentrations of 1–6 mM.

### Forming droplet interface bilayers

Droplet interface bilayers (DIBs) were formed in computer numerical control (CNC)-machined (Roland monoFab SRM-20) 3 × 3 well arrays made from poly(methyl methacrylate) (PMMA) (Supplementary Fig. 10). Typically, 200 µL of lipid–oil solution was added to the well array followed by injection (using a 0.5 µL Hamilton syringe) of two 75 nL PBS droplets into each well. The droplets were left separate in the lipid–oil solution for 15 min to complete monolayer formation at the oil–water interface. Following this, droplets were brought together using gravity by tilting the well array. Once a DIB had formed, it was left for an hour to reach the equilibrium contact angle (*θ*_DIB_).

The same protocol was applied to form droplet assemblies comprising two-dimensional (2D) triplets and three-dimensional (3D) tetrahedrons, with the exception of manually positioning droplets by using a thin plastic wire. Temperature experiments were performed using a Tokai Hit^TM^ Leica TPX (Type HF) Thermo Plate. Contact angle measurements were taken after one hour at a set temperature. Droplet pairs were kept hydrated during the hour of heating by using wet paper towels to maintain a saturated humidity and prevent evaporation of the aqueous droplets. Droplets of 100 µm in diameter (0.52 nL in volume) were generated using the 3D printer.

### Imaging the change in contact angle over time

A DIB was formed between two droplets (≈75 nL) in CNC-machined PMMA wells that consisted of two levels (at different heights) connected by a slope with an angle of 17° (Supplementary Fig. 10b). One droplet was positioned at the bottom of the track, while the other was formed at the top. By using a pipette tip, the top droplet was pushed to roll down the track so that it came into contact with the bottom droplet. The increase in contact angle over time, once the two droplets made contact, was imaged by optical microscopy (HCX PL FLUOTAR 10×/0.30na, brightfield at 10 frames s^−1^). Experiments were run for a maximum of 20 min after two droplets had made contact.

### Preparing quartz cuvettes

Custom quartz cuvettes were purchased from Starna Scientific at dimensions of 20 × 10 × 10 mm (*x, y, z*). Cuvettes were used for surface studies and as a container for 3D-printing droplet networks. Cuvettes were cleaned by sonicating in 5% (v/v) Decon 90 in Milli-Q water and ethanol for 15 min (with plenty of rinsing with Milli-Q water in between and as the last step). Cuvettes were treated in an O_2_ plasma cleaner for 4 min before printing and surface studies. Roughened cuvettes were achieved by incubating the cuvettes in 8 M NaOH for 7 days.

### 3D-printing droplet networks

Our custom droplet 3D printer is extensively outlined in previous work^4^. In brief, a piezoelectric transducer transmits controlled pressure impulses to a chamber filled with Milli-Q water. The chamber is connected to a glass nozzle (with a tip diameter of approximately 150 µm) from which the aqueous printing solution is ejected. To prevent mixing of the printing solution with the water inside the chamber and nozzle, an undecane oil plug of ≈5 µL was used to separate the two solutions. The nozzle tip was immersed into the lipid–oil solution contained in the quartz cuvette, which was positioned on a digitally controlled micromanipulator (PatchStar micromanipulator, Scientifica, 20 nm resolution). The micromanipulator movements and the piezo actuation were synchronised using a custom-made software developed in LabView. Printing was monitored using a side-on stereomicroscope (Nikon SMZ745T), and videos and pictures were acquired using a digital camera (Thorlabs DCC1645C) mounted on the microscope.

### Imaging droplet networks

Pairs and small networks of droplets were imaged using N PLAN 5×/0.12, 10× or HCX PL FUOTAR 20×/0.40 dry objectives on a Leica DMi8 inverted epi-fluorescence microscope in brightfield or fluorescence mode using an excitation lamp at 450–490 nm with an emission cut-off at 550 nm (exciting Atto488). Printed networks were imaged using a Leica SP5 confocal microscope using a HC PL Fluotar 10×/0.30 objective (0.3 numerical aperture), at an excitation wavelength of 546 nm and emission cut-off at 625 nm (exciting Atto550M), a *z*-step of 8 µm and scanning speed of 100 Hz.

### Imaging and reconstructing hydrogel droplet replicas

Hydrogel replicas of droplets within the networks were used to reconstruct their 3D shape^38^. A photo-cross-linkable pre-hydrogel solution was used as the aqueous phase comprising 20% (w/v) poly(ethylene glycol) diacrylate (M_n_ = 700 g mol^−1^, Sigma-Aldrich), 0.5% (w/v) Irgacure 2959 (Sigma-Aldrich) as the photo-initiator, and 100 µM ethidium bromide-*N,N’*-bisacrylamide (Sigma-Aldrich) as the cross-linkable fluorophore. The lipid–oil solution was composed of 1 mM DPhPC dissolved in an undecane/silicone oil solution at a silicone oil volume fraction (*φ*_SIL_) of 0.61 (corresponding to a calculated *θ*_DIB_ of 37.3° from Supplementary Fig. 5b). Networks (10 × 12 × 4; *x*, *y*, *z*,) with droplet diameters of 80–100 µm were printed. Since oxygen is a strong inhibitor of free radical polymerisation^39^, the networks were purged in N_2_ for at least 1 h before photo-polymerisation. Photo-polymerisation was initiated by illuminating the networks under a UV lamp (COP5-A Nikon Eclipse, Thorlabs) for 7 min. After hydrogel formation, DPhPC was precipitated by exchanging the printing lipid–oil solution by serial dilution with 100% silicone oil. Gelled networks were then transferred to PBS solutions by adding PBS to the networks and removing the oil solution. During this process, the cross-linked hydrogel networks break apart into individual droplets or small clusters of polyhedral microgels that retain the geometry of the droplets within the 3D-printed network.

The droplet replicas were imaged using an Olympus Fluoview FV3000 inverted microscope with a UPlanSApo 20×/0.75 dry objective. An excitation wavelength of 405 nm and emission cut-off between 420 and 520 nm (exciting the cross-linked ethidium bromide) was used. Images were sampled according to the Nyquist criterion in *x*, *y* and *z* dimensions. 3D shapes of the polyhedral microgel clusters were reconstructed and rendered using Arivis Vision4D image analysis software (Fig. 5).

### Measuring contact angles

The contact angle formed between two droplets (Fig. 1a) was calculated from the radii of each droplet (*R*_1_, *R*_2_), and the centre-to-centre distance (*L*) by using the formula^40^ (Supplementary Fig. 1a):

2 $$2\theta _{\mathrm{DIB}} = {\mathrm{cos}}^{ - 1}\left( {\frac{{L^2 - R_1^2 - R_2^2}}{{2R_1R_2}}} \right)$$

Care was taken to produce 75 nL droplets each time as the observed centre-to-centre distance would be inaccurate if the radii were not the same.

The contact angle of droplets formed with the surface (*θ*_surface_) was calculated using Supplementary Eq. 5 from the radius of the bilayer formed with the surface (*R*_b_) and the radius of the droplet (spherical cap) (*R*_a_) (Supplementary Fig. 7a):

3 $$\theta _{{\mathrm{surface}}} = {\mathrm{sin}}^{ - 1}\left( {\frac{{R_{\mathrm{b}}}}{{R_{\mathrm{a}}}}} \right)$$

Contact angles were calculated from microscopy images using a custom-written script on MATLAB^®^ (Mathworks, Natick, MA) to measure relevant radii and centre-to-centre distances.

### Classifying printed network regions

To quantify statistics of the droplet networks, a 2D plane from the confocal stack of each 3D network was manually selected to maximise the fluorescent signal from the lipid bilayers between droplets of the bottom layer of each print. This image was then segmented into separate droplets and oil inclusions, initially by a ridge detection algorithm^41^ and then by manual verification and cleaning. The resulting segmented networks were analysed using MATLAB^®^ (Mathworks, Natick, MA). In the first stage, we classified contiguous regions of the network as either droplets or oil inclusions. This was done on a morphological basis: droplet areas were required to be between 0.4 and 10 times the area of the median region, and to have a face area to convex hull area ratio of at least 0.9. This second condition ensured that only regions that were largely convex were counted as droplets. Other regions were classified as oil inclusions. Droplet excess in the first layer (the number of droplets that fell from the upper layers to the first layer) of the networks was calculated from (*N*−*N*_*t*_)/*N*_*t*_, where $N$ is the number of counted droplets in the first layer and $N_t$ is the number of expected droplets in the first layer (Supplementary Fig. 2a–c). $N_t$ = 72, when a perfectly packed network of 7 × 8 × 4 (*x*, *y*, *z*) droplets is formed; the first layer commonly appeared with a dimension of 8 × 9 (*x*, *y*) droplets because of droplets falling from the second and third layer (Supplementary Fig. 2a–c). The variation in droplet size (coefficient of variation) was calculated from the standard deviation of droplet area within a network divided by the average droplet area.

Bias in network classification was avoided by performing manual network adjustments blind to the experimental conditions used in each print.

### Classifying packing types

To estimate the proportion of packing types within each network, we first generated a mesh over the print by performing a Delaunay triangulation using the centres of all regions classified as droplets as the input points. A convenient property of this triangulation method is that the circumcircle of each triangle contains no other points; each triangle therefore represents a triplet of neighbouring droplets and provides localised geometrical information about their arrangements.

The plot of the bivariate distribution of the largest angle of each triangle ($\theta _{{\mathrm{max}}}$) vs the triangle area normalised by the average droplet area ($\hat A$) for all prints revealed two distinct clusters of triangles, one corresponding to hexagonally packed droplet regions ($\theta _{{\mathrm{max}}} \approx 60^\circ$), and one corresponding to square-packed droplet regions ($\theta _{{\mathrm{max}}} \approx 90^\circ$) (Supplementary Fig. 3d). Reasoning that these represented stable configurations of droplets for certain printing parameters, we classified each triangle using these clusters. Four packing categories were used: ‘hexagonal’ (closely packed triplets of droplets arranged as equilateral triangles), ‘square’ (closely packed triplets of droplets arranged as right-angled triangles), ‘amorphous’ (closely packed triplets of droplets of intermediate arrangement) and ‘no packing’ (triplets of droplets that are not close-packed). For a hexagonal classification, $60^\circ \le \theta _{{\mathrm{max}}} < 67^\circ$. For an amorphous classification, $67^\circ \le \theta _{{\mathrm{max}}} < 83^\circ$. For a square classification, $83^\circ \le \theta _{{\mathrm{max}}} < 97^\circ$. In addition, for all of these classes, $0.25 < \hat A < 0.75$. Triangles that fell outside of these ranges were classified as no packing (Supplementary Fig. 3d).

Some elongated ‘sliver’ triangles were classified as being a member of one of the closely packed classes, despite being composed of widely spaced droplets. Closer analysis of these triangles revealed that they were generally composed of two closely packed droplets and a single more distant droplet, together forming a skinny near-isosceles triangle. We therefore applied an additional constraint based on the perimeter of each triangle normalised by the average droplet radius ($\hat P$). Since for perfect hexagonal packing $\hat P = 3\sqrt 3$, and for a perfect square packing $\hat P = 4 + 2\sqrt 2$, we set the constraint $\hat P < 3.73 + 0.0545\;\theta _{{\mathrm{max}}}$ for the closely packed classes based on a linear interpolation between these two points. Our chosen *y*-intercept for this constraint ($3.73$) is slightly greater than the theoretical *y*-intercept from the interpolation, allowing triangles with small deviations from the perfect packing geometry to be classified as closely packed while still ensuring classification of sliver triangles as no packing.

### Localisation of packing types in printed networks

For the dataset with maximal hexagonal packing (*φ*_SIL_ = 0.55 and *x*_POPC_ = 0.13), 2D network images were manually registered to a reference network image using the Interactive Rigid Transform plugin for ImageJ. This plugin only permits rotational and translational transformations, so the geometry of the network is preserved during registration. Regions of registered images were then classified as droplets or inclusions, and droplet triplet triangles were extracted and classified as described above. For each print $j$ and each of the four packing types $i$, binary images $B({\boldsymbol{R}})_{i,j}$ were generated indicating the presence or absence of each packing type at each spatial location ${\boldsymbol{R}}$. A rough heatmap for each class was then calculated as the sum of the binary images over all prints, $H({\boldsymbol{R}})_i = \mathop {\sum }\limits_j B({\boldsymbol{R}})_{i,j}$.

To create the regularised heatmaps (Fig. 4b), an idealised version of the print geometry was manually registered to the reference network image. The boundaries of the droplets in this registered idealised image were used to define a set of hexagonal bins *h*, associated with a collection of pixel locations ${\boldsymbol{r}}_h$. For each bin, the sum of all pixels within its boundaries for each of the four rough heatmaps $s_{h,i} = \mathop {\sum }\limits_{{\boldsymbol{r}}_h} H({\boldsymbol{R}})_i$ was then calculated. Finally, the normalised packing proportion $\hat s_{h,i}$ for each bin and each packing type was calculated as $\hat s_{h,i} = s_{h,i}/\mathop {\sum }\limits_i s_{h,i}$.

### Electrophysiological recordings

For the electrophysiological recordings shown in Fig. 6, a lipid–oil solution composed of 2 mM DPhPC in an undecane-silicone oil mixture with *φ*_SIL_ = 0.43 was used. The aqueous solution used for both conductive and non-conductive droplets was 25 mM Tris-HCl pH 7.6, 1 M NaCl, 0.1% (w/v) Pluronic F68. In the droplets that would form the conductive pathway, alpha hemolysin (αHL) purified from *Staphylococcus* *aureus* (Wood 46, ATCC) was added at a final concentration of 60 µg/mL in the same aqueous solution.

The recording electrodes were assembled by soldering silver wires (100 µm diameter, Sigma-Aldrich) to male crimp connectors. Before each recording session, the tips of the silver wires were incubated in sodium hypochlorite solution (Sigma-Aldrich) for at least 10 min and coated with a thin layer of 1.5% (w/v) agarose (ultra-low gelling temperature agarose, Sigma-Aldrich) in the same aqueous solution as described above.

The electrophysiological recordings were performed inside a Faraday cage (Mechanical Workshop, University of Oxford) which fitted the headstage of a patch-clamp amplifier (Axopatch 200B, Axon Instruments). The Ag/AgCl electrodes were plugged into female crimp connectors fixed on two micromanipulators (Narishige, NMN-1). The female crimp connectors were electrically connected to the headstage of the amplifier. By operating the micromanipulators, the Ag/AgCl electrode tips were positioned so that they would touch the ends of the αHL-containing droplet pathways in the printed networks. The electrical traces were recorded with pClamp 10 (Molecular Devices) and processed with MATLAB^®^.

## Supplementary information

Supplementary Information

Description of Additional Supplementary Files

Supplementary Movie 1

Supplementary Movie 2

## Data Availability

Raw confocal images and processed images used to study the packing in droplet networks can be found at 10.5061/dryad.k0p2ngf4z. Our custom-written scripts can be found at https://github.com/Pseudomoaner/NetPack. All other relevant data are available from the corresponding authors upon reasonable request.
