# Supplementary material for: Controlled packing and single-droplet resolution of 3D-printed functional synthetic tissues
Source: Nat Commun. 2020 Apr 30;11:2105. doi: 10.1038/s41467-020-15953-y (PMC7192927; doi:10.1038/s41467-020-15953-y)
Supplement: Supplementary file 1 — Supplementary Information [file 41467_2020_15953_MOESM1_ESM.pdf]

Supplementary Information

Controlled packing and single-droplet resolution of 3D-printed functional synthetic tissues

Alessandro Alcinesio, Oliver J. Meacock, Rebecca G. Allan, Carina Monico, Vanessa Restrepo Schild, Idil Cazimoglu, Matthew Cornall, Ravinash Krishna Kumar and Hagan Bayley

Supplementary Figures

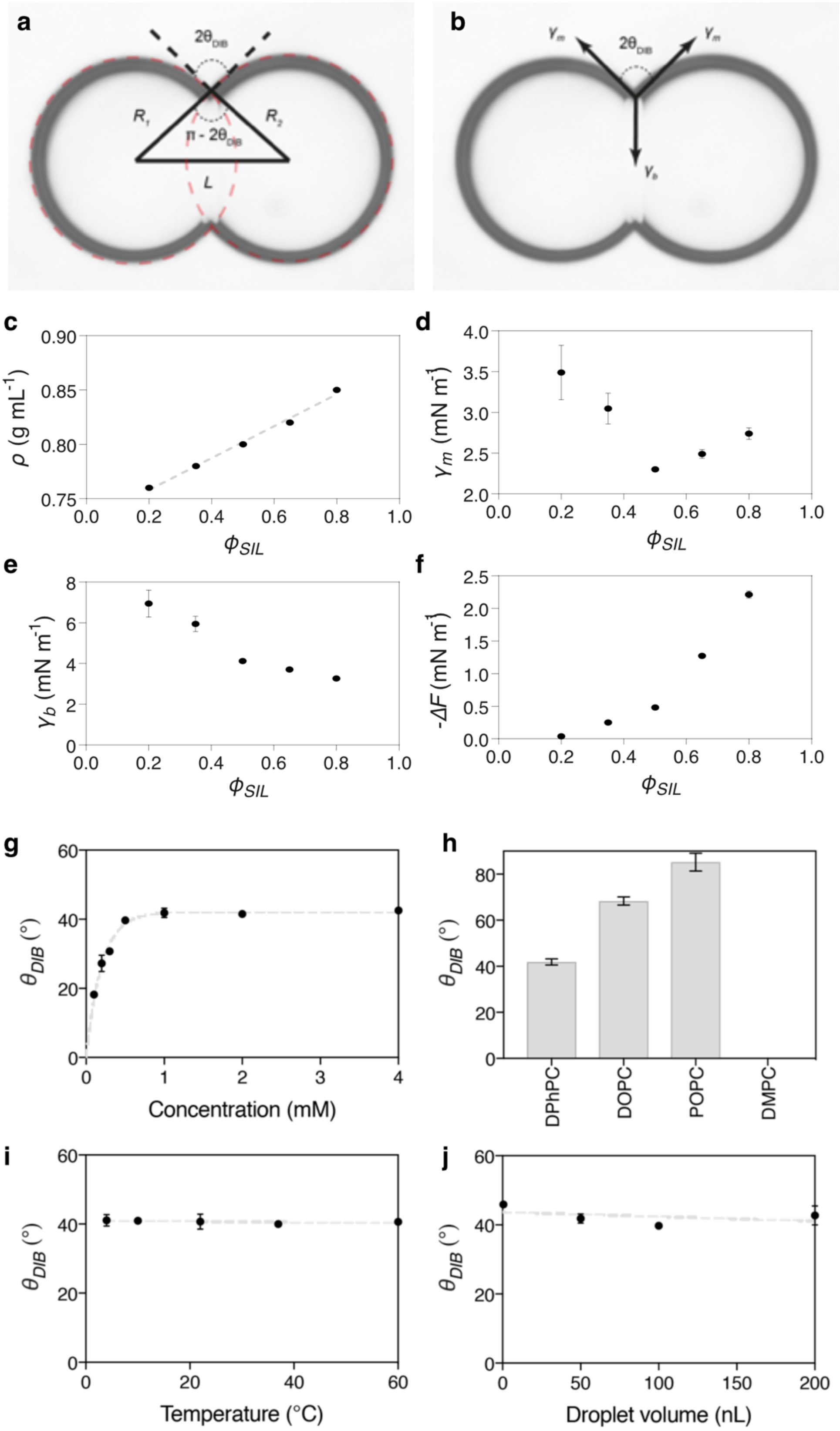

Supplementary Fig. 1. a-b, Overlays on optical microscopy images of droplet pairs (1 mM DPhPC, ϕ_{SIL} = 0.65) describing how θ_{DIB} was calculated (**a**), and how the bilayer and monolayer surface tensions (γ_b and γ_m) act on a DIB (**b**). **c**, A plot of the

relationship between ϕ_{SIL} and the density (ρ) of the oil solution. ρ is directly proportional to ϕ_{SIL} ($R^2 = 0.99$). **d**, A plot of the relationship of the monolayer interfacial tension (γ_m) and ϕ_{SIL} as measured from pendant drop analysis. **e**, A plot of the relationship between the bilayer interfacial tension (γ_b) and ϕ_{SIL} in a DIB formed between two 75 nL aqueous droplets. γ_b was calculated from Supplementary Eq. 2 from measured γ_m and θ_{DIB} values. **f**, A plot of the relationship of the change in energy of adhesion per unit area (ΔF) and ϕ_{SIL} in a DIB formed between two 75 nL aqueous droplets. ΔF was calculated from Supplementary Eq. 3 using measured γ_m and θ_{DIB}. **g**, The dependence of θ_{DIB} with respect to DPhPC concentration when $\phi_{SIL} = 0.65$. The nonlinear regression in grey is a single decay exponential ($R^2 = 0.97$) (see Supplementary Table 4). The concentration where the slope plateaus was determined at $y = 41.6°$, which was 1% below the plateau value of 42.0°. This yielded a concentration of 0.91 mM. **h**, The dependence of θ_{DIB} with respect to lipid composition. The concentration of lipid was 1 mM and $\phi_{SIL} = 0.65$. With DMPC, DIBs did not form; $n = 6$ pairs immediately coalesced when brought together. **i**, The dependence of θ_{DIB} with respect to temperature (1 mM DPhPC and $\phi_{SIL} = 0.65$). Droplet pairs were formed and maintained in a hydrated environment for 1 hour at a constant temperature. The linear regression slope in grey shows insignificant deviation from zero (one-way ANOVA, $F(4, 18) = 0.76$, p-value = 0.32) (see Supplementary Table 3). **j**, The dependence of θ_{DIB} with respect to droplet volume (1 mM DPhPC and $\phi_{SIL} = 0.65$). Droplet pairs with diameters of 100 μm (0.52 nL) were generated using the droplet printer. The linear regression slope in grey insignificantly deviates from zero (p-value = 0.57) (see Supplementary Table 5). In **c**-**j** a minimum of $n = 3$ repeats was used for each measurement, each point is the mean value, and the error bars are the standard deviation.

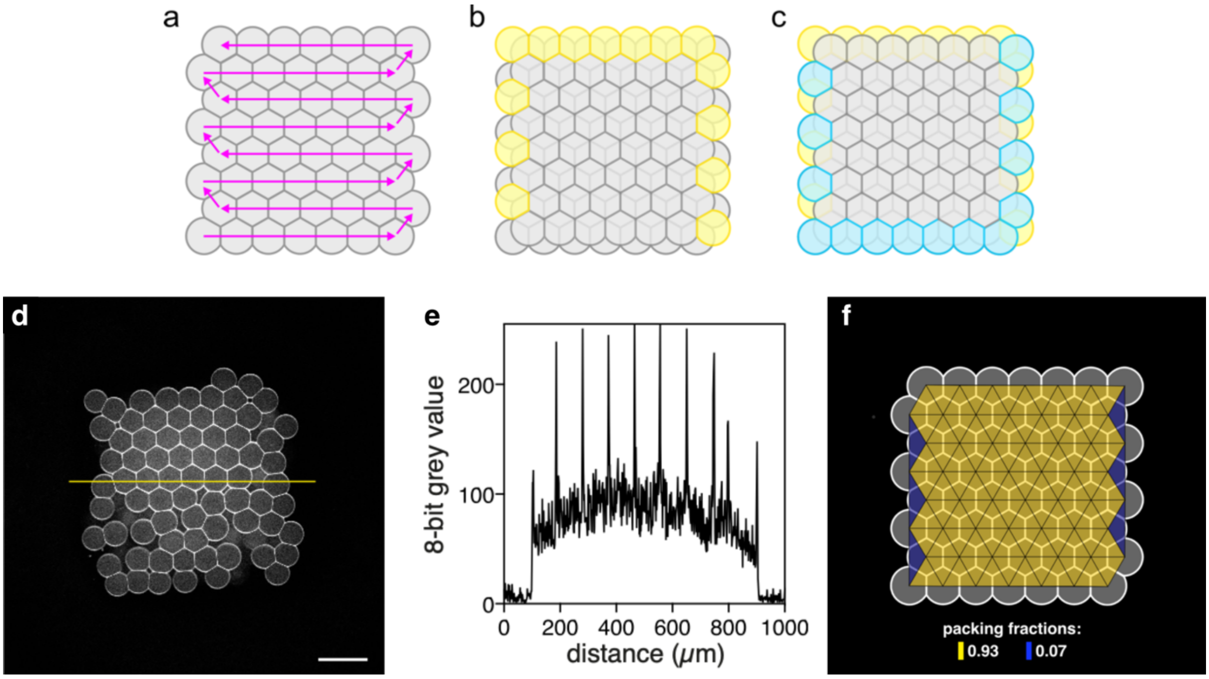

Supplementary Fig. 2. a, An idealised diagram of the first layer of a 7 x 8 x 4 (x, y, z) 3D-printed droplet network with the printing path overlaid. Droplets are positioned in a line-by-line fashion from the bottom left to the top left. **b** and **c**, idealised diagrams of the second (**b**), and the second and third (**c**) layers overlaid on the first layer. Edge droplets from the second (yellow) and third (cyan) often fell to the lower layers because of insufficient support from droplets below (supported by one or two droplets). Theoretically, considering these fallen droplets form the upper layers from a perfectly hexagonally close-packed lattice, the first layer would contain 8 x 9 (x, y) droplets, rather than 7 x 8 (x, y) droplets as shown in **a**. **d**, A confocal microscopy image of a horizontal cross-section of layer 1 (bottom) in a 7 x 8 x 4 (x, y, z) 3D-printed droplet network. Conditions used were 1 mM DPhPC and ϕ_{SIL} = 0.59. Scale bar = 200 μm. **e**, The corresponding plot of the line intensity profile (yellow line in **d**), demonstrating the localisation of Atto550M at the monolayers and bilayers of the droplet network from corresponding peaks of high grey values (near 255 for an 8-bit image) along the line profile. **f**, The packing types analysis based on Delaunay triangulation of an ideal map of the first layer in printed networks results in a hexagonal packing fraction of 0.93 and of a no-packing fraction of 0.07. These values were used to normalise the quantification of all reported packing fractions.

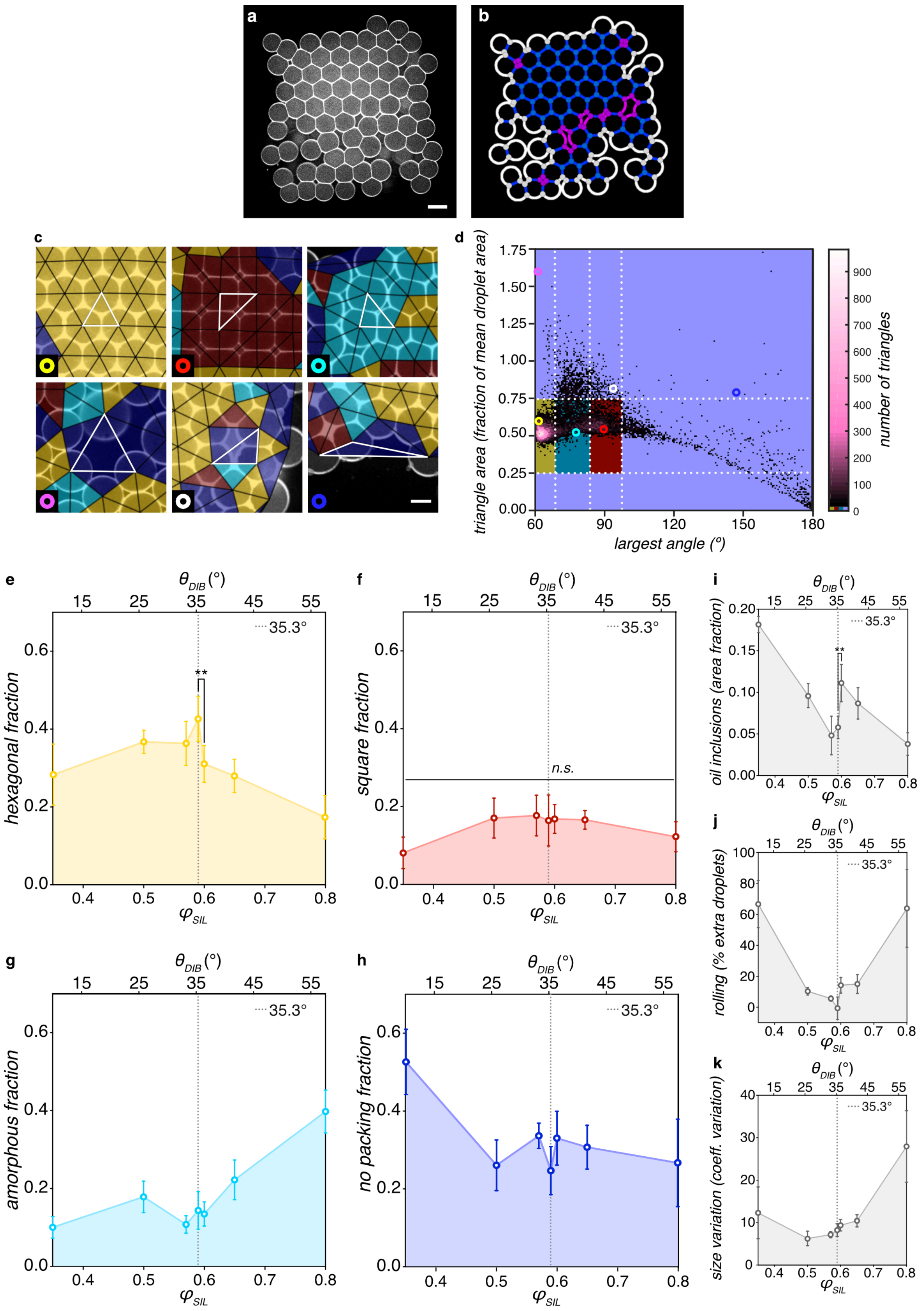

Supplementary Fig. 3. a, Confocal fluorescent microscopy picture of the first layer of a 3D-printed droplet. Lipid bilayers and monolayers were visualised with Atto550M. **b**, A processed image of **a** showing monolayers (white), bilayers (blue), and oil inclusions (magenta). **c**, Examples of different packing types are indicated by white triangles on

confocal images of layer 1. Our classification method draws triangles (indicated by black and white highlighted triangles) between the centres of three neighbouring droplets and assigns the local packing type of each triplet based on the triangle geometry (Delaunay triangulation). Droplet triplets are classified as packed in a hexagonal (yellow), square (red), or amorphous (cyan) fashion, when the largest angle of the triangle is between 60 and 67°, 83 and 97°, or 67° and 83°, respectively. Normalised triangle areas and perimeters are also used as constraints in our classification method (see Supplementary Methods). Triangles outside these limits were assigned to the no-packing type (blue). **d**, A heat map of common packing types derived from over 129 networks. The colour of each bin indicates the local density of triangles in the bivariate distribution of maximum interior angles and normalised areas. The shaded areas in yellow, red, cyan and blue correspond to hexagonal, square, amorphous, and no packing, respectively. Coloured circles correspond to the specific examples shown in **c** (highlighted by white triangles). Scale bars are 100 μm for **a** and 50 μm for **c**. **e-h**, Plots of hexagonal (**e**), square (**f**), amorphous (**g**), and no-packing (**h**) area fractions in the first layer of 3D-printed droplet networks at increasing values of ϕ_{SIL}. **i-k**, Plots of area fractions of oil inclusions (**i**), droplet rolling (**j**), and droplet size variation (**k**) with ϕ_{SIL}. In plots **e**-**k**: each point is the mean from $n > 3$ networks (for individual n values see Supplementary Table 21), error bars represent the standard deviation, and the data were analysed by using One-Way ANOVA with Tuckey's multiple comparisons test (see Supplementary Tables 11-14); stars indicate the following significance levels, **: *p-value* < 0.01, *: *p-value* < 0.05 and *n.s.*: *p-value* > 0.05; θ_{DIB} was calculated from Eq. 1; the dashed grey line marks the critical angle (θ_c) of 35.3°; the lines between the points and the shaded areas underneath the lines were not mathematically fitted.

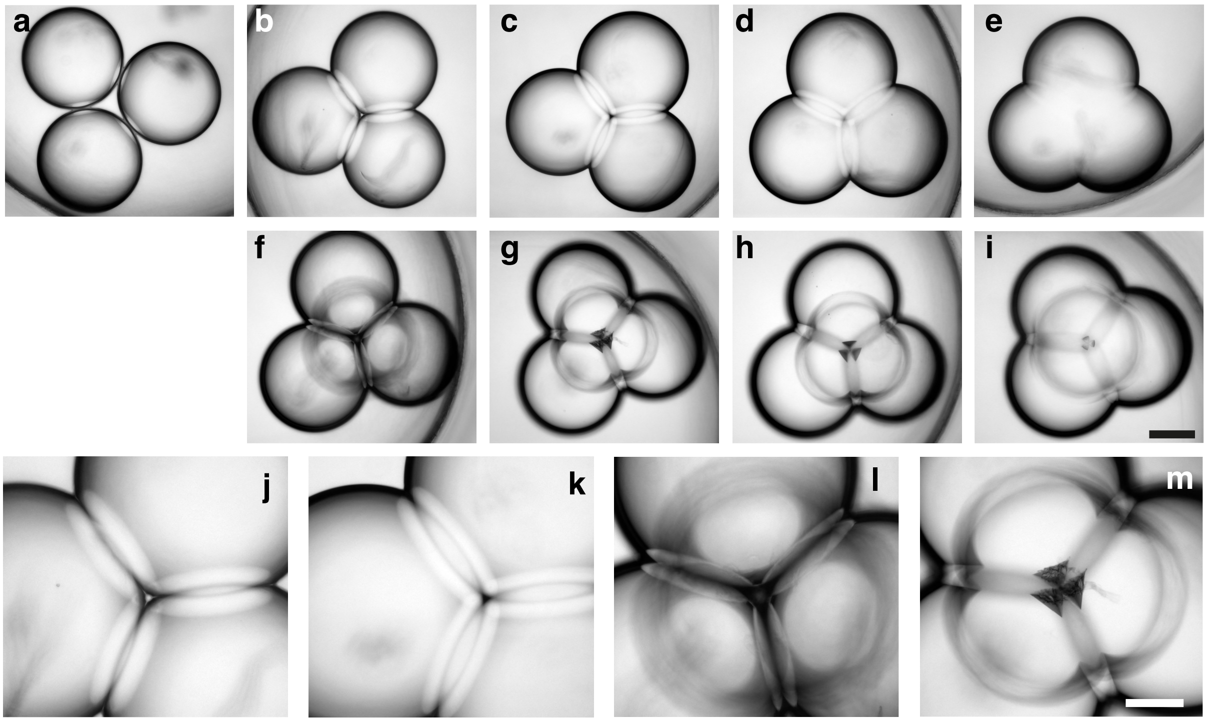

Supplementary Fig. 4. **a**-**m**, Brightfield microscopy images of triplets of droplets arranged where their centres lie at the corners of an equilateral triangle, and equivalent quartets of droplets arranged where their centres lie at the corners of a regular tetrahedron at $\phi_{SIL} = 0.2$ ($\theta_{DIB} = 6.0 \pm 0.7°$) (**a**), $\phi_{SIL} = 0.5$ ($\theta_{DIB} = 26.5 \pm 1.7°$) (**b** and **f**), $\phi_{SIL} = 0.6$ ($\theta_{DIB} = 36.6°$, calculated from Fig. 1h) (**c** and **g**), $\phi_{SIL} = 0.65$ ($\theta_{DIB} = 41.9 \pm 1.3°$) (**d** and **h**), and $\phi_{SIL} = 0.8$ ($\theta_{DIB} = 53.4 \pm 0.8°$) (**e** and **i**). **j** and **k** are higher magnification images of **b** and **c** triplets. **i** and **m** are higher magnification images of **f** and **g**. **j**–**k** and **l**–**m** demonstrate the macroscopic closing of the trigonal and tetrahedral borders, respectively. 1 mM DPhPC was used in every image. Scale bars in **i** and **m** are 200 μm and 100 μm, respectively.

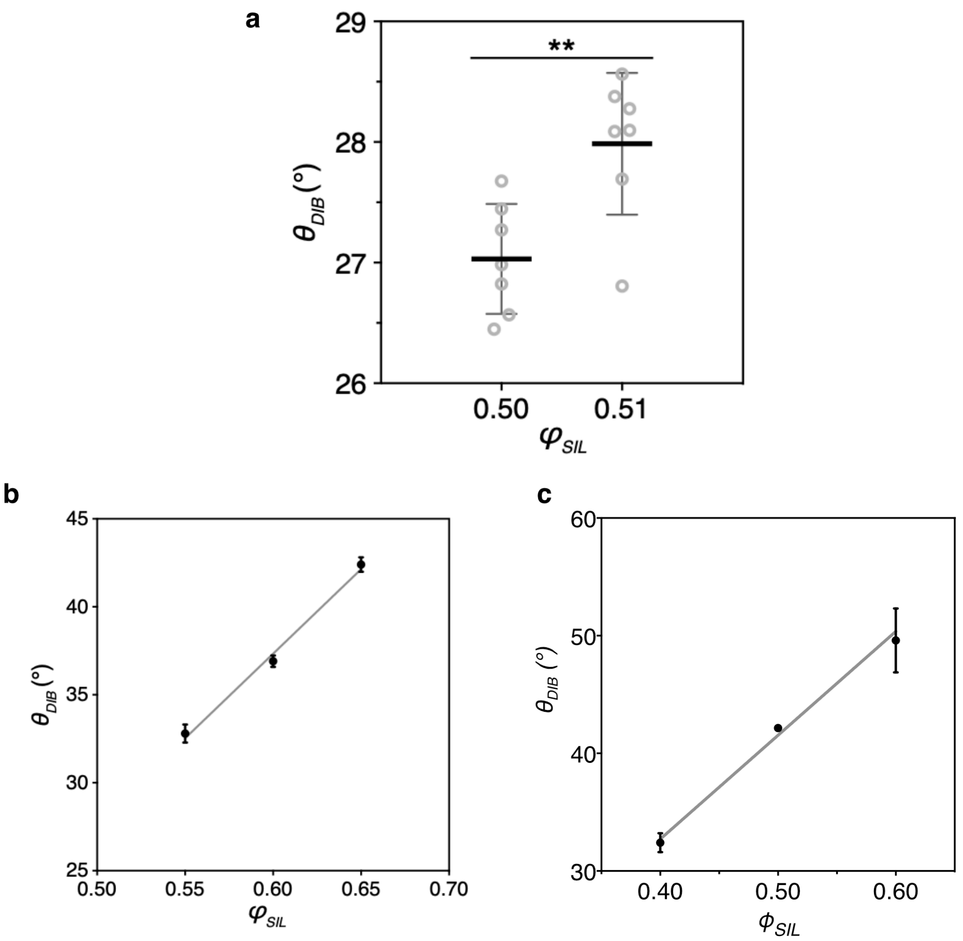

Supplementary Fig. 5. a, A comparison of θ_{DIB} measurements in droplet pairs formed at ϕ_{SIL} = 0.50 and ϕ_{SIL} = 0.51. The increase in ϕ_{SIL} caused a significant increase in θ_{DIB} from 27.0 ± 0.5° to 28.0 ± 0.6° (unpaired t-test with Welch correction, p-value < 0.01, n = 7 for both conditions, see Supplementary Table 20). The black lines are the mean value of n = 7 replicates (values shown as grey circles) per condition and error bars are the standard deviation. **b**, A plot for showing the linear dependence of θ_{DIB} with ϕ_{SIL} at a lipid composition of 1 mM DPhPC and aqueous solution comprising PBS, 20% (w/v) poly(ethylene glycol) diacrylate, and 0.5% (w/v) Irgacure 2959 (R^2 = 0.98, see Supplementary Table 10). **c**, A plot for showing the linear dependence of θ_{DIB} with ϕ_{SIL} at a lipid composition of 2 mM DPhPC and aqueous solution composed of 25 mM Tris-HCl pH 7.6, 1M NaCl and 0.1 wt% Pluronic F68 (R^2 = 0.97, see Supplementary Table 25).

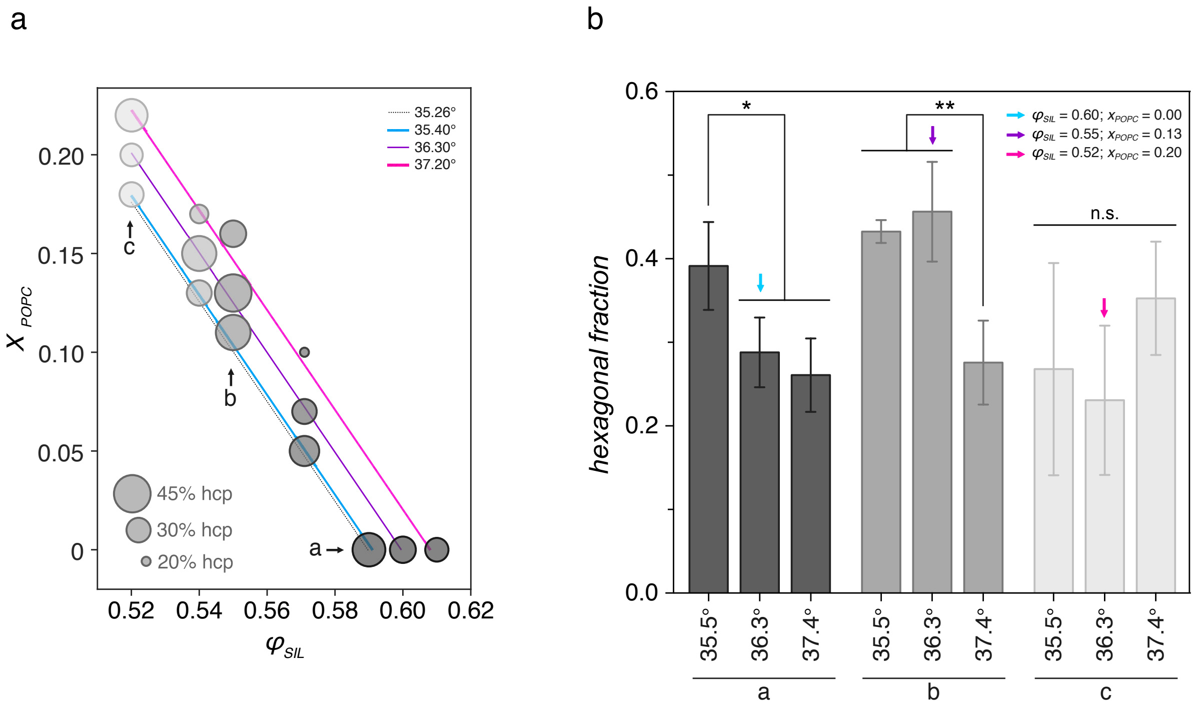

Supplementary Fig. 6. a, The relationship of the area fraction of hexagonal packing in the first layer of 3D-printed droplet networks with respect to ϕ_{SIL} and x_{POPC}. The size of points indicates the percentage of area occupied by hexagonal packing in the first layer. Coloured lines indicate conditions with the same θ_{DIB} value. Groups a, b, and c, (color-coded with different shades of grey) indicate conditions grouped and shown in **b**. **b**, A bar graph demonstrating changes in the hexagonal packing fraction in the first layer of 3D-printed droplet networks with regards to groups a, b, and c shown in **a**. Coloured arrows indicate conditions used at the same θ_{DIB} value. See Supplementary Tables 17, 18 and 19 for statistical tests and Supplementary Table 22 for individual values. See Supplementary Table 11 for a complete list of conditions used.

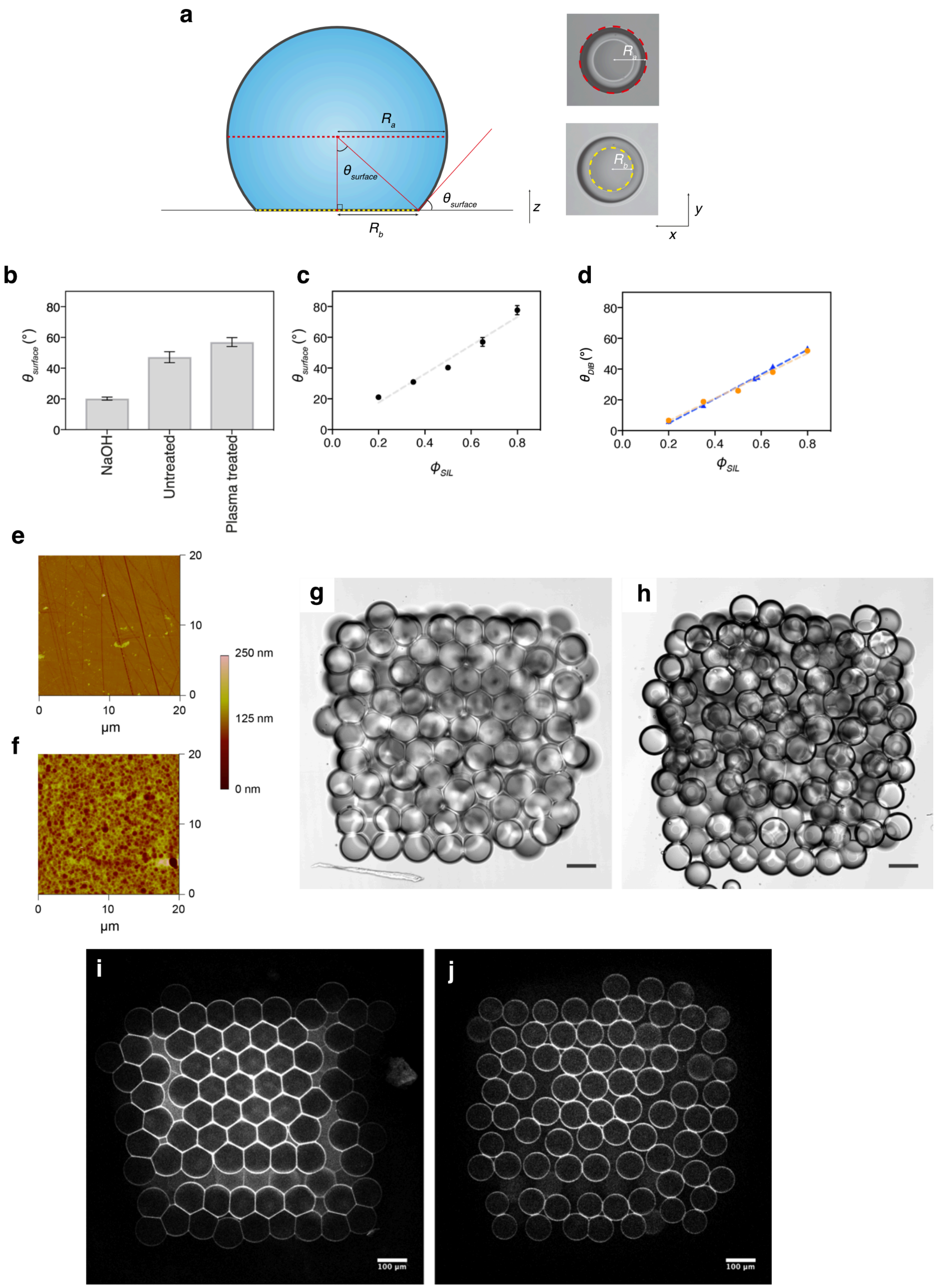

Supplementary Fig. 7. a, A schematic demonstrating how $\theta_{surface}$ was calculated. Optical microscopy images show where R_a and R_b were measured with respect to the cross-section in the schematic. **b**, A bar graph of the dependence of $\theta_{surface}$ with respect to treated surface (ϕ_{SIL} = 0.65 and 1 mM DPhPC). **c**, A plot demonstrating the

linear dependence of $\theta_{surface}$ with respect to ϕ_{SIL} ($R^2 = 0.96$) (see Supplementary Table 7 for statistical tests). **d**, A comparison plot of θ_{DIB} between droplet pairs formed on PMMA (blue) and those formed on plasma-cleaned quartz (orange) at different ϕ_{SIL}. Linear regressions were fitted to each data set ($R^2 = 0.99$ for PMMA and $R^2 = 0.97$ for plasma-cleaned quartz). The two regressions were not significantly different (*F*-test, *p-value* = 0.6740) (see Supplementary Table 6 for statistical tests). For **b-d**, 1 mM DPhPC was used. A minimum of $n = 3$ repeats was used for each measurement. The points are the mean and the error bars are the standard deviation; and 1 mM DPhPC was used in every condition. **e**-**f,** Atomic force microscopy images of untreated quartz (**e**) and NaOH-treated quartz (**f**). In **e** and **f,** the difference in roughness is clearly observed. Scale bar on the right represents the height in **e** and **f**. **g**-**h**, Brightfield microscopy images of 3D-printed droplet networks formed on O_2 plasma-treated quartz (**g**) and roughened quartz (NaOH treatment) (**h**). In **g** and **h**, the lipid and oil composition were1 mM DPhPC and $\phi_{SIL} = 0.59$, respectively. **i**-**j**, Horizontal cross-sections acquired by confocal microscopy of the first layers in 7 x 8 x 4 (*x*, *y*, *z*) 3D droplet networks printed at relative humidities of 45% (**i**) and 32% (**j**). In **i** and **j**, the lipid and oil composition were 1 mM DPhPC and $\phi_{SIL} = 0.59$, respectively.

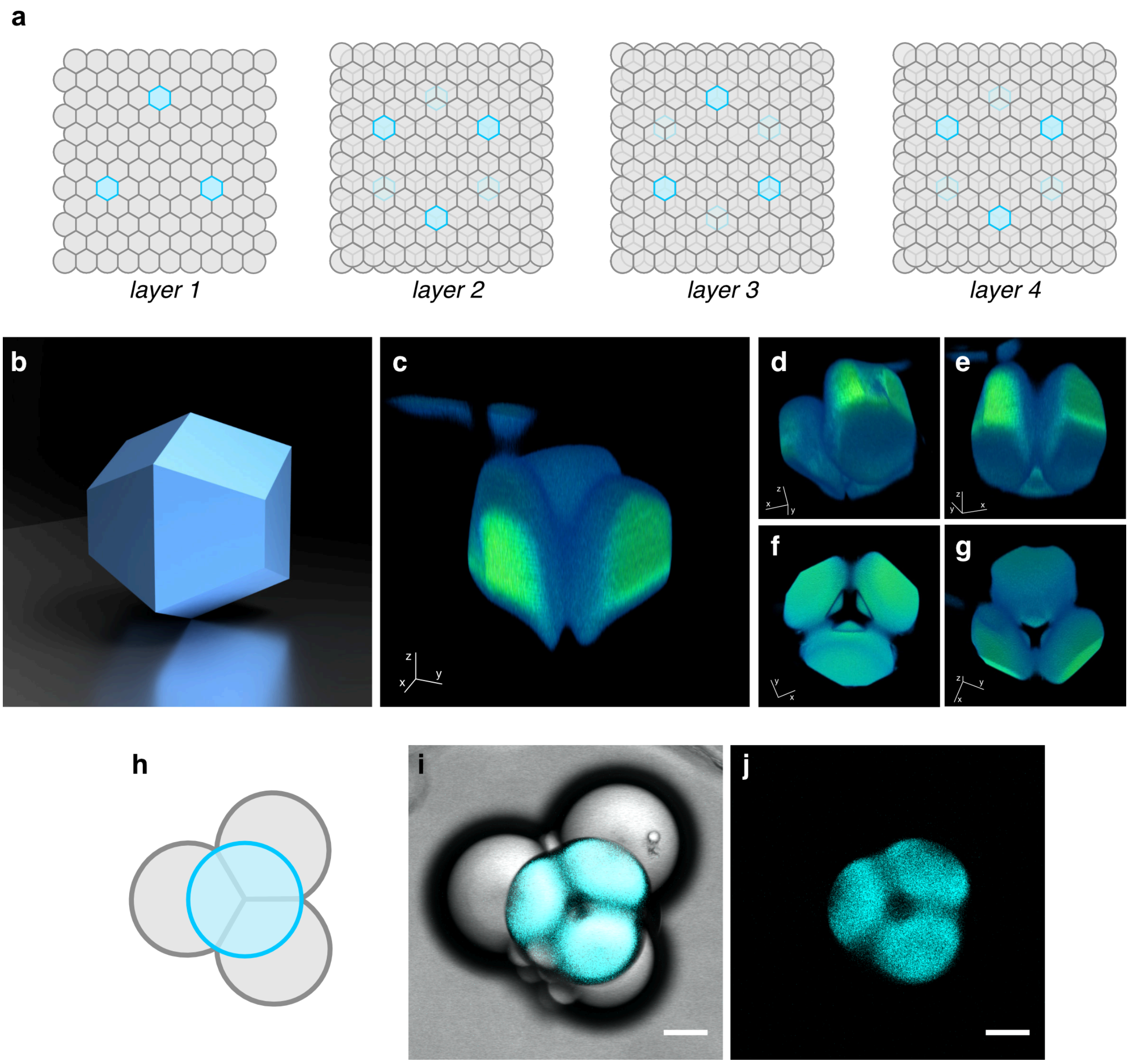

Supplementary Fig. 8. a, A schematic of a 10 x 12 x 4 (*x*, *y*, *z*) droplet network showing the positioning of fluorescently labelled (by 10 μm Atto 448) droplets in layers 1 (**i**), 2 (**ii**), 3 (***iii***), and 4 (***iv***). **b-g**, Reconstructed z-stacks from confocal microscopy images of a fluorescently labelled droplet in layer 2 surrounded by 12 droplets. **b** is a computer model of the theoretical space-filling shape of a droplet in a droplet network arranged in a hexagonally close-packed fashion, and **c-g** are the corresponding reconstructed images of the droplet shape at different orientations. In **f**, the three-fold symmetry is observed at the bottom of the assumed trapezo-rhombic dodecahedron droplet (as compared to **b**), but the optical aberrations caused by the difference in refractive indices between the oil and aqueous phases prevented further imaging of the above six-fold and three-fold symmetries. **h-j**, An example of the observed optical aberrations in 3D droplet networks. **h** is a schematic of four 75 nL-droplets arranged where their centres lie at the corners of a regular tetrahedron. The top droplet contains 10 μm calcein. **i** is the composite brightfield and fluorescent image and **j** is the fluorescent image of the top droplets; both were acquired by epifluorescence microscopy. Scale bars in **i** and **j** are 150 μm.

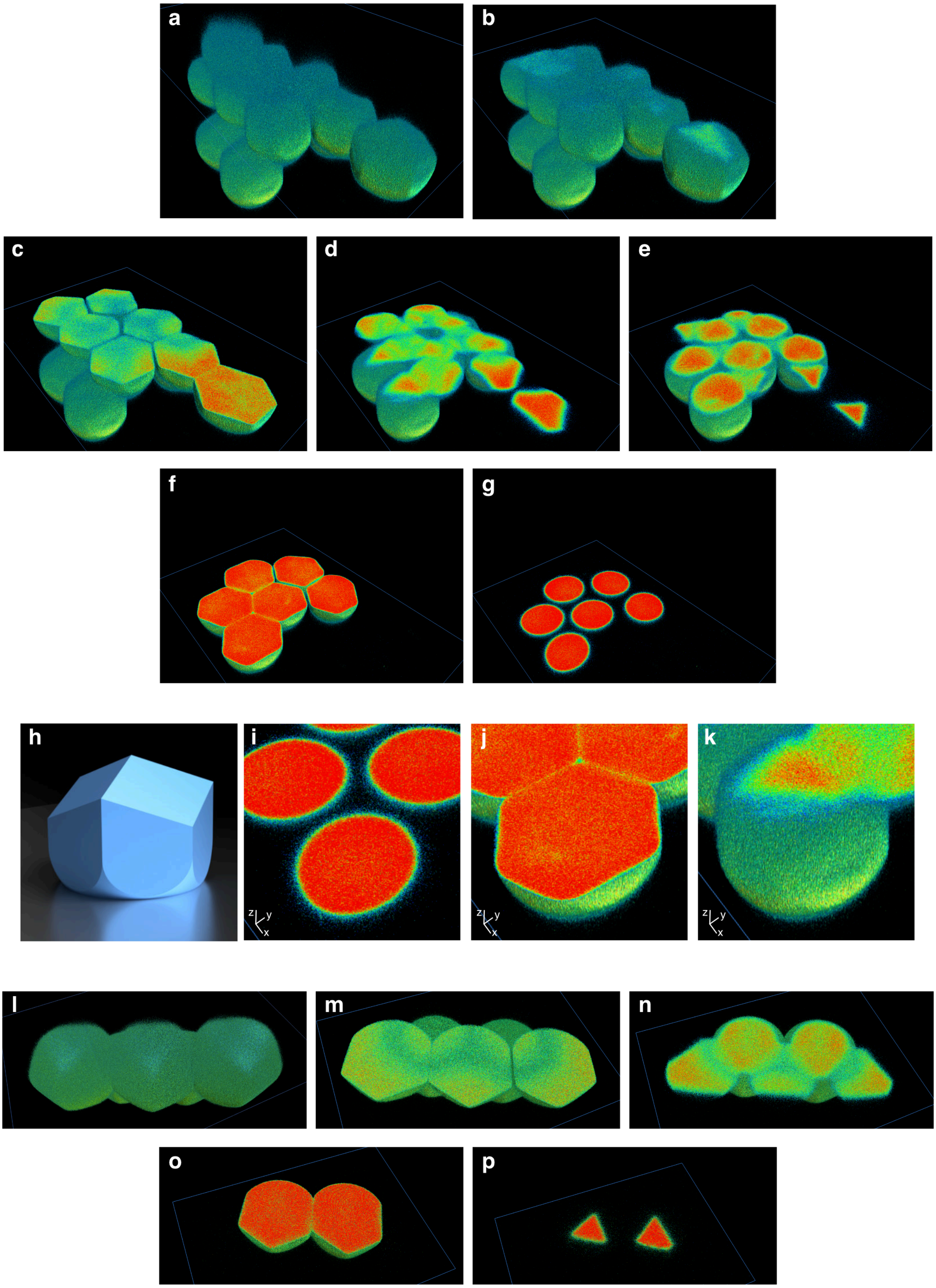

Supplementary Fig. 9. a-g, 3D reconstruction generated from confocal images of a hexagonally close-packed cluster of connected polyhedral hydrogels re-dispersed in PBS. **a-g**, Images are *z*-sections from the top of the third layer to the bottom of the first layer. **h**, A computer model of the space-filling shape of droplets hexagonally

packed in the first layer – a truncated 10-faced polyhedron with C_{3v} symmetry. **i-k**, *z*-sectioning from the top of a droplet in the first layer going from a circular base (**i**) to six-fold symmetry (**j**) to a three-fold symmetry (**k**). The truncation of the droplet shape is because of contact with the flat quartz surface. **l-p**, *z*-sectioning of the space-filling shape of edge droplets in the printed network. Incomplete polyhedra with one curved face at the oil-lipid-water interface can be seen.

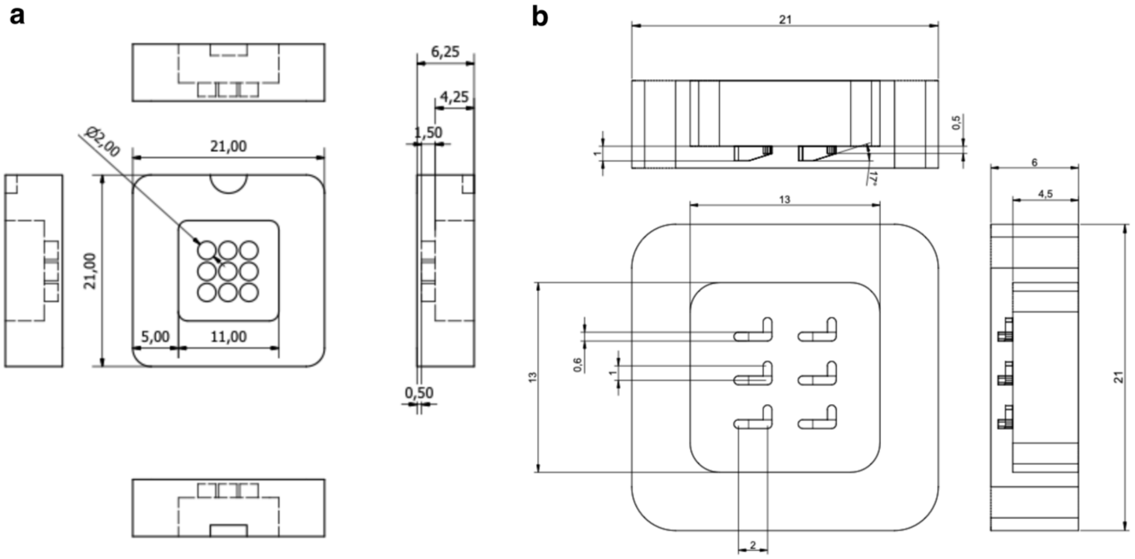

Supplementary Fig. 10. a-**b**, Technical drawings of a 3 x 3 well array used to screen the contact angle in droplet pairs (**a**) and of a well array used for monitoring the kinetics of DIB formation overtime. The well arrays were designed in Autodesk AutoCAD and manufactured in poly(methyl methacrylate) plastic using a desktop milling machine (Roland monoFab SRM-20). Sizes are in mm.

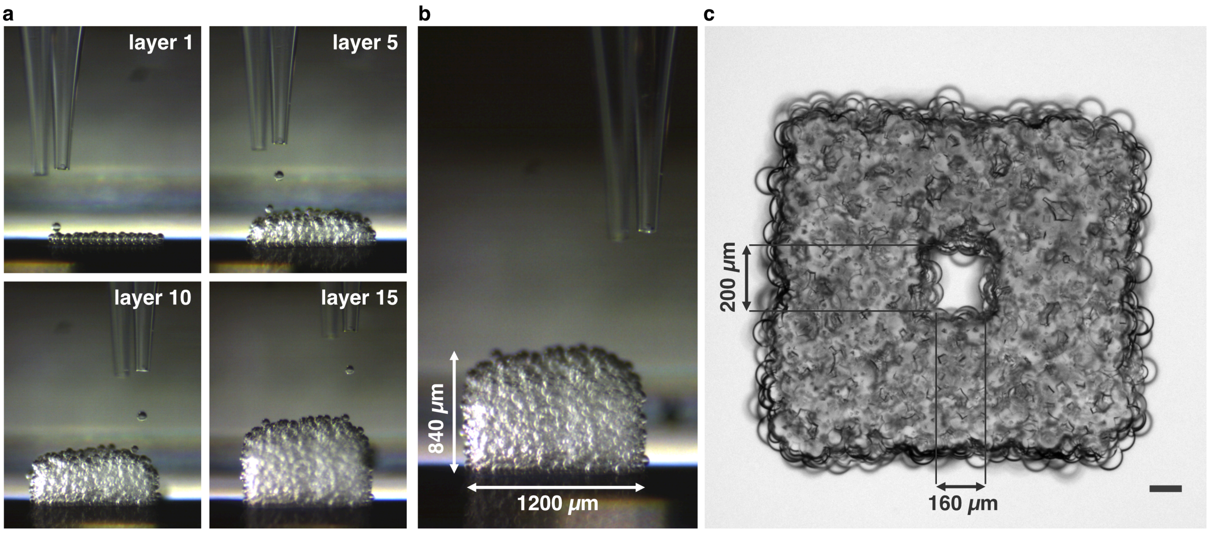

Supplementary Fig. 11. 3D printing of a droplet network (dimensions 12 x 13 x 15 droplets in *x*, *y* and *z* directions) with a hollow channel (dimensions 4 x 5 x 15

droplets in x, y and z directions) at its centre. **a**, Side view images of the droplet network during printing of the first, fifth, tenth, and fifteenth layers. **b**, Side view of the printed network, measuring approximately 1200 μm in width and 840 μm in height. **c**, Brightfield image of the network containing a hollow channel of dimensions 160 x 200 x 840 μm in x, y and z directions.

Supplementary Tables

Supplementary Table 1: Dependence of θ_{DIB} on ϕ_{SIL} – Linear regression (Fig. 1f)

Linear Regression	
Slope	86.26 ± 5.704
Y-intercept when X=0.0	-13.70 ± 3.139
X-intercept when Y=0.0	0.1589
1/slope	0.01159
95% Confidence Intervals	
Slope	70.43 to 102.1
Y-intercept when X=0.0	-22.42 to -4.989
X-intercept when Y=0.0	0.06967 to 0.2233
Goodness of Fit	
R square	0.9828
Sy.x	2.742
Is slope significantly non-zero?	
F	228.7
DFn, DFd	1.000, 4.000
P value	0.0001
Deviation from zero?	Significant
Data	
Number of X values	6
Maximum number of Y replicates	1
Total number of values	6
Number of missing values	0
Equation	Y = 86.26*X - 13.70

Supplementary Table 2: Dependence of θ_{DIB} on x_{POPC} – Linear regression (Fig. 1g)

Linear Regression	
Slope	45.61 ± 1.773
Y-intercept when X=0.0	39.56 ± 1.041
X-intercept when Y=0.0	-0.8674
1/slope	0.02193
95% Confidence Intervals	
Slope	41.41 to 49.80

Y-intercept when X=0.0	37.10 to 42.02
X-intercept when Y=0.0	-1.009 to -0.7492
Goodness of Fit	
R square	0.9895
Sy.x	1.644
Is slope significantly non-zero?	
F	661.4
DFn, DFd	1.000, 7.000
P value	< 0.0001
Deviation from zero?	Significant
Data	
Number of X values	9
Maximum number of Y replicates	1
Total number of values	9
Number of missing values	0
Equation	Y = 45.61*X + 39.56

Supplementary Table 3: Dependence of θ_{DIB} on temperature – one-way ANOVA (Supplementary Fig. 1i)

ANOVA table	**SS**	**DF**	**MS**	**F (DFn, DFd)**	**P value**
Treatment (between columns)	3.937	4	0.9841	F (4, 18) = 0.7648	P = 0.5618
Residual (within columns)	23.16	18	1.287		
Total	27.10	22			

Tukey's multiple comparisons test	**Mean Diff.**	**95% CI of diff.**	**Significant?**	**Summary**
4.00 vs. 10.00	0.1259	-2.044 to 2.295	No	ns
4.00 vs. 22.00	0.3910	-2.114 to 2.896	No	ns
4.00 vs. 37.00	1.081	-0.9958 to 3.158	No	ns
4.00 vs. 60.00	0.4236	-1.877 to 2.725	No	ns
10.00 vs. 22.00	0.2651	-2.240 to 2.770	No	ns
10.00 vs. 37.00	0.9554	-1.122 to 3.033	No	ns
10.00 vs. 60.00	0.2976	-2.003 to 2.599	No	ns
22.00 vs. 37.00	0.6903	-1.735 to 3.116	No	ns
22.00 vs. 60.00	0.03252	-2.587 to 2.652	No	ns
37.00 vs. 60.00	-0.6578	-2.872 to 1.556	No	ns

Supplementary Table 4: Dependence of θ_{DIB} on lipid concentration – non-linear regression (Supplementary Fig. 1g)

One phase decay	
Best-fit values	
Y0	= 0.0
Plateau	42.04
K	5.037

Half Life	0.1376
Tau	0.1985
Span	= -42.04
Std. Error	
Plateau	0.3978
K	0.1927
95% Confidence Intervals	
Plateau	41.22 to 42.86
K	4.640 to 5.434
Half Life	0.1276 to 0.1494
Tau	0.1840 to 0.2155
Goodness of Fit	
Degrees of Freedom	25
R square	0.9700
Absolute Sum of Squares	55.70
Sy.x	1.493
Constraints	
Y0	Y0 = 0.0
K	K > 0.0
Number of points	
Analyzed	27

Supplementary Table 5: Dependence of θ_{DIB} on droplet volume – linear regression (Supplementary Fig. 1j)

Linear Regression	
Slope	-0.01174 ± 0.005849
Y-intercept when X=0.0	41.70 ± 0.6701
X-intercept when Y=0.0	3553
1/slope	-85.22
95% Confidence Intervals	
Slope	-0.03690 to 0.01343
Y-intercept when X=0.0	38.82 to 44.58
X-intercept when Y=0.0	1188 to +infinity
Goodness of Fit	
R square	0.6680
Sy.x	0.8620
Is slope significantly non-zero?	
F	4.025
DFn, DFd	1.000, 2.000
P value	0.1827
Deviation from zero?	Not Significant
Data	
Number of X values	4
Maximum number of Y replicates	1

Total number of values	4
Number of missing values	0
Equation	Y = -0.01174*X + 41.70

Supplementary Table 6: Dependence of θ_{DIB} on the surface – comparison between PMMA and plasma-cleaned quartz (Supplementary Fig. 7d)

Linear Regression	θ_{DIB} (on PMMA)	θ_{DIB} (on glass)
Slope	79.88 ± 3.169	73.27 ± 4.318
Y-intercept when X=0.0	-11.28 ± 1.744	-8.395 ± 2.345
X-intercept when Y=0.0	0.1412	0.1146
1/slope	0.01252	0.01365
95% Confidence Intervals		
Slope	71.08 to 88.67	59.53 to 87.01
Goodness of Fit		
R square	0.9937	0.9897
Sy.x	1.523	2.048
Is slope significantly non-zero?		
F	635.4	287.9
DFn, DFd	1.000, 4.000	1.000, 3.000
P value	< 0.0001	0.0004
Deviation from zero?	Significant	Significant
Data		
Number of X values	6	5
Maximum number of Y replicates	1	1
Total number of values	6	5
Number of missing values	0	1
Equation	Y = 79.88*X - 11.28	Y = 73.27*X - 8.395
Comparison of Slopes		
F	1.59061	
DFn, DFd	1, 7	
P value	0.2476	
Significant (alpha=0.05)?	No	
Pooled slope	76.618	
Comparison of intercepts		
F	0.172938	
DFn, DFd	1, 8	
P value	0.6884	
Significant (alpha=0.05)?	No	
Pooled intercept	-9.81533	

Supplementary Table 7: Contact angle on quartz glass – comparison between $\theta_{surface}$ and θ_{DIB} formed on glass (Supplementary Fig. 7c)

Linear Regression	θ_{DIB} (on glass)	$\theta_{surface}$
Slope	73.27 ± 4.318	92.88 ± 9.672
Y-intercept when X=0.0	-8.395 ± 2.345	-1.052 ± 5.253
X-intercept when Y=0.0	0.1146	0.01133
1/slope	0.01365	0.01077
95% Confidence Intervals		
Slope	59.53 to 87.01	62.10 to 123.7
Goodness of Fit		
R square	0.9897	0.9685
Sy.x	2.048	4.588
Is slope significantly non-zero?		
F	287.9	92.21
DFn, DFd	1.000, 3.000	1.000, 3.000
P value	0.0004	0.0024
Deviation from zero?	Significant	Significant
Data		
Number of X values	5	5
Maximum number of Y replicates	1	1
Total number of values	5	5
Number of missing values	1	1
Equation	Y = 73.27*X - 8.395	Y = 92.88*X - 1.052
Comparison of Slopes		
F	3.42628	
DFn, DFd	1, 6	
P value	0.1136	
Significant (alpha=0.05)?	No	
Pooled slope	83.0767	
Comparison of intercepts		
F	43.2398	
DFn, DFd	1, 7	
P value	0.0003	
Significant (alpha=0.05)?	Yes	

Supplementary Table 8: Dependence of θ_{DIB} on the ϕ_{SIL} *and* x_{POPC} – 2D linear regression (Fig. 1h)

2D Linear Regression	
Equation	Z = (a*X + b*Y + c) / d
a	0.930
b	0.368
c	-0.236
d	0.009
R squared	0.9897

Supplementary Table 9: Lipid/oil composition for Fig. 3 and Supplementary Fig. 6

	φ_{SIL}	x_{POPC}	θ_{DIB} *(predicted)*
a	0.52	0.18	35.43
	0.52	0.20	36.26
	0.52	0.22	37.10
	0.54	0.13	35.45
	0.54	0.15	36.28
	0.54	0.17	37.12
b	0.55	0.11	35.67
	0.55	0.13	36.50
	0.55	0.16	37.76
	0.57	0.05	35.42
	0.57	0.07	36.26
	0.57	0.10	37.51
c	0.59	0.00	35.29
	0.60	0.00	36.35
	0.61	0.00	37.41

Supplementary Table 10: Dependence of θ_{DIB} on the ϕ_{SIL} – PEGDA DIBs (Supplementary Fig. 5b)

Linear Regression	
Slope	96.08 ± 3.817
Y-intercept when X=0.0	-20.31 ± 2.295
X-intercept when Y=0.0	0.2114
1/slope	0.01041
95% Confidence Intervals	
Slope	87.67 to 104.5
Y-intercept when X=0.0	-25.36 to -15.26
X-intercept when Y=0.0	0.1740 to 0.2428
Goodness of Fit	
R square	0.9829
Sy.x	0.5397
Is slope significantly non-zero?	
F	633.7
DFn, DFd	1.000, 11.00
P value	< 0.0001
Deviation from zero?	Significant
Data	
Number of X values	3
Maximum number of Y replicates	5
Total number of values	13
Number of missing values	2
Equation	Y = 96.08*X - 20.31

Supplementary Table 11: Hexagonal fraction – one-way ANOVA for Supplementary Fig. 3e

ANOVA table	**SS**	**DF**	**MS**	**F (DFn, DFd)**	**P value**
Treatment (between columns)	0.1965	6	0.03276	F (6, 28) = 9.615	P < 0.0001
Residual (within columns)	0.09539	28	0.003407		
Total	0.2919	34			

Tukey's multiple comparisons test	**Mean Diff.**	**95% CI of diff.**	**Significant?**	**Summary**
0.35 vs. 0.50	-0.07464	-0.2099 to 0.06058	No	ns
0.35 vs. 0.57	-0.06895	-0.2042 to 0.06626	No	ns
0.35 vs. 0.59	-0.1270	-0.2580 to 0.003887	No	ns
0.35 vs. 0.60	0.02453	-0.1107 to 0.1597	No	ns
0.35 vs. 0.65	0.005412	-0.1298 to 0.1406	No	ns
0.35 vs. 0.80	0.1001	-0.03080 to 0.2310	No	ns
0.50 vs. 0.57	0.005688	-0.1114 to 0.1228	No	ns
0.50 vs. 0.59	-0.05239	-0.1645 to 0.05972	No	ns
0.50 vs. 0.60	0.09917	-0.01793 to 0.2163	No	ns
0.50 vs. 0.65	0.08005	-0.03705 to 0.1972	No	ns

0.50 vs. 0.80	0.1748	0.06265 to 0.2869	Yes	***
0.57 vs. 0.59	-0.05808	-0.1702 to 0.05403	No	ns
0.57 vs. 0.60	0.09348	-0.02362 to 0.2106	No	ns
0.57 vs. 0.65	0.07436	-0.04274 to 0.1915	No	ns
0.57 vs. 0.80	0.1691	0.05696 to 0.2812	Yes	***
0.59 vs. 0.60	0.1516	0.03945 to 0.2637	Yes	**
0.59 vs. 0.65	0.1324	0.02033 to 0.2446	Yes	*
0.59 vs. 0.80	0.2272	0.1203 to 0.3341	Yes	****
0.60 vs. 0.65	-0.01912	-0.1362 to 0.09798	No	ns
0.60 vs. 0.80	0.07559	-0.03652 to 0.1877	No	ns
0.65 vs. 0.80	0.09471	-0.01740 to 0.2068	No	ns

Supplementary Table 12: Square fraction – one-way ANOVA for Supplementary Fig. 3f

ANOVA table	**SS**	**DF**	**MS**	**F (DFn, DFd)**	**P value**
Treatment (between columns)	0.02826	6	0.004710	F (6, 28) = 2.149	P = 0.0789
Residual (within columns)	0.06136	28	0.002192		
Total	0.08962	34			

Tukey's multiple comparisons test	**Mean Diff.**	**95% CI of diff.**	**Significant?**	**Summary**
0.35 vs. 0.50	-0.08942	-0.1979 to 0.01903	No	ns
0.35 vs. 0.57	-0.09579	-0.2042 to 0.01266	No	ns
0.35 vs. 0.59	-0.08307	-0.1881 to 0.02194	No	ns
0.35 vs. 0.60	-0.08702	-0.1955 to 0.02143	No	ns
0.35 vs. 0.65	-0.08486	-0.1933 to 0.02359	No	ns
0.35 vs. 0.80	-0.04125	-0.1463 to 0.06376	No	ns
0.50 vs. 0.57	-0.006374	-0.1003 to 0.08755	No	ns
0.50 vs. 0.59	0.006345	-0.08358 to 0.09627	No	ns
0.50 vs. 0.60	0.002394	-0.09153 to 0.09631	No	ns
0.50 vs. 0.65	0.004553	-0.08937 to 0.09847	No	ns
0.50 vs. 0.80	0.04817	-0.04175 to 0.1381	No	ns
0.57 vs. 0.59	0.01272	-0.07720 to 0.1026	No	ns
0.57 vs. 0.60	0.008768	-0.08515 to 0.1027	No	ns
0.57 vs. 0.65	0.01093	-0.08299 to 0.1048	No	ns
0.57 vs. 0.80	0.05454	-0.03538 to 0.1445	No	ns
0.59 vs. 0.60	-0.003952	-0.09387 to 0.08597	No	ns
0.59 vs. 0.65	-0.001793	-0.09171 to 0.08813	No	ns
0.59 vs. 0.80	0.04182	-0.04392 to 0.1276	No	ns
0.60 vs. 0.65	0.002159	-0.09176 to 0.09608	No	ns
0.60 vs. 0.80	0.04577	-0.04415 to 0.1357	No	ns
0.65 vs. 0.80	0.04361	-0.04631 to 0.1335	No	ns

Supplementary Table 13: No-pack fraction – one-way ANOVA for Supplementary Fig. 3h

ANOVA table	SS	DF	MS	F (DFn, DFd)	P value
Treatment (between columns)	0.2054	6	0.03424	F (6, 28) = 6.594	P = 0.0002
Residual (within columns)	0.1454	28	0.005192		
Total	0.3508	34			

Tukey's multiple comparisons test	Mean Diff.	95% CI of diff.	Significant?	Summary
0.35 vs. 0.50	0.2422	0.07529 to 0.4091	Yes	**
0.35 vs. 0.57	0.1721	0.005219 to 0.3391	Yes	*
0.35 vs. 0.59	0.2535	0.09189 to 0.4151	Yes	***
0.35 vs. 0.60	0.09672	-0.07021 to 0.2636	No	ns
0.35 vs. 0.65	0.2014	0.03446 to 0.3683	Yes	*
0.35 vs. 0.80	0.2384	0.07681 to 0.4001	Yes	**
0.50 vs. 0.57	-0.07007	-0.2146 to 0.07449	No	ns
0.50 vs. 0.59	0.01130	-0.1271 to 0.1497	No	ns
0.50 vs. 0.60	-0.1455	-0.2901 to -0.0009405	Yes	*
0.50 vs. 0.65	-0.04083	-0.1854 to 0.1037	No	ns
0.50 vs. 0.80	-0.003788	-0.1422 to 0.1346	No	ns
0.57 vs. 0.59	0.08137	-0.05704 to 0.2198	No	ns
0.57 vs. 0.60	-0.07543	-0.2200 to 0.06913	No	ns
0.57 vs. 0.65	0.02924	-0.1153 to 0.1738	No	ns
0.57 vs. 0.80	0.06629	-0.07212 to 0.2047	No	ns
0.59 vs. 0.60	-0.1568	-0.2952 to -0.01839	Yes	*
0.59 vs. 0.65	-0.05213	-0.1905 to 0.08628	No	ns
0.59 vs. 0.80	-0.01508	-0.1471 to 0.1169	No	ns
0.60 vs. 0.65	0.1047	-0.03989 to 0.2492	No	ns
0.60 vs. 0.80	0.1417	0.003307 to 0.2801	Yes	*
0.65 vs. 0.80	0.03705	-0.1014 to 0.1755	No	ns

Supplementary Table 14: Oil inclusions – one-way ANOVA for Supplementary Fig. 3i

ANOVA table	SS	DF	MS	F (DFn, DFd)	P value
Treatment (between columns)	0.05593	6	0.009321	F (6, 28) = 30.86	P < 0.0001
Residual (within columns)	0.008457	28	0.0003020		
Total	0.06438	34			

Tukey's multiple comparisons test	Mean Diff.	95% CI of diff.	Significant?	Summary
0.35 vs. 0.50	0.08539	0.04513 to 0.1257	Yes	****
0.35 vs. 0.57	0.1334	0.09312 to 0.1736	Yes	****
0.35 vs. 0.59	0.1236	0.08463 to 0.1626	Yes	****
0.35 vs. 0.60	0.07034	0.03008 to 0.1106	Yes	***
0.35 vs. 0.65	0.09480	0.05454 to 0.1351	Yes	****
0.35 vs. 0.80	0.1436	0.1047 to 0.1826	Yes	****
0.50 vs. 0.57	0.04799	0.01312 to 0.08285	Yes	**
0.50 vs. 0.59	0.03823	0.004846 to 0.07161	Yes	*

0.50 vs. 0.60	-0.01505	-0.04991 to 0.01982	No	ns
0.50 vs. 0.65	0.009409	-0.02546 to 0.04428	No	ns
0.50 vs. 0.80	0.05825	0.02487 to 0.09163	Yes	***
0.57 vs. 0.59	-0.009759	-0.04314 to 0.02362	No	ns
0.57 vs. 0.60	-0.06303	-0.09790 to -0.02817	Yes	****
0.57 vs. 0.65	-0.03858	-0.07345 to -0.003711	Yes	*
0.57 vs. 0.80	0.01026	-0.02312 to 0.04364	No	ns
0.59 vs. 0.60	-0.05327	-0.08666 to -0.01989	Yes	***
0.59 vs. 0.65	-0.02882	-0.06220 to 0.004563	No	ns
0.59 vs. 0.80	0.02002	-0.01181 to 0.05185	No	ns
0.60 vs. 0.65	0.02445	-0.01041 to 0.05932	No	ns
0.60 vs. 0.80	0.07329	0.03991 to 0.1067	Yes	****
0.65 vs. 0.80	0.04884	0.01546 to 0.08222	Yes	**

Supplementary Table 15: Hexagonal fraction – one-way ANOVA for Fig. 3c

ANOVA table	**SS**	**DF**	**MS**	**F (DFn, DFd)**	**P value**
Treatment (between columns)	0.1170	2	0.05850	F (2, 10) = 15.74	P = 0.0008
Residual (within columns)	0.03716	10	0.003716		
Total	0.1542	12			

Tukey's multiple comparisons test	**Mean Diff.**	**95% CI of diff.**	**Significant?**	**Summary**
cyan vs. purple	-0.1683	-0.2740 to -0.06258	Yes	**
cyan vs. magenta	0.05737	-0.06467 to 0.1794	No	ns
purple vs. magenta	0.2256	0.1036 to 0.3477	Yes	**

Supplementary Table 16: Hexagonal fraction – one-way ANOVA for Fig. 3d (purple)

ANOVA table	**SS**	**DF**	**MS**	**F (DFn, DFd)**	**P value**
Treatment (between columns)	0.09743	2	0.04872	F (2, 9) = 15.04	P = 0.0013
Residual (within columns)	0.02915	9	0.003239		
Total	0.1266	11			

Tukey's multiple comparisons test	**Mean Diff.**	**95% CI of diff.**	**Significant?**	**Summary**
0.25 vs. 0.5	-0.1564	-0.2724 to -0.04033	Yes	*
0.25 vs. 2	0.04136	-0.08000 to 0.1627	No	ns
0.5 vs. 2	0.1977	0.09114 to 0.3043	Yes	**

Supplementary Table 17: Hexagonal fraction – one-way ANOVA for Supplementary Fig. 6b (x_{POPC} = 0.00, dark grey bars)

ANOVA table	**SS**	**DF**	**MS**	**F (DFn, DFd)**	**P value**
Treatment (between columns)	0.06295	2	0.03148	F (2, 12) = 11.50	P = 0.0016
Residual (within columns)	0.03283	12	0.002736		
Total	0.09579	14			

Tukey's multiple comparisons test	Mean Diff.	95% CI of diff.	Significant?	Summary
35.4° vs. 36.3°	0.1160	0.03147 to 0.2005	Yes	**
35.4° vs. 37.4°	0.1474	0.05727 to 0.2374	Yes	**
36.3° vs. 37.4°	0.03138	-0.06224 to 0.1250	No	ns

Supplementary Table 18: Hexagonal fraction – one-way ANOVA for Supplementary Fig. 6b (ϕ_{SIL} = 0.55, grey bars)

ANOVA table	SS	DF	MS	F (DFn, DFd)	P value
Treatment (between columns)	0.1137	2	0.05685	F (2, 10) = 18.40	P = 0.0004
Residual (within columns)	0.03089	10	0.003089		
Total	0.1446	12			

Tukey's multiple comparisons test	Mean Diff.	95% CI of diff.	Significant?	Summary
35.4° vs. 36.3°	-0.02494	-0.1362 to 0.08633	No	ns
35.4° vs. 37.4°	0.1757	0.06438 to 0.2869	Yes	**
36.3° vs. 37.4°	0.2006	0.1042 to 0.2970	Yes	***

Supplementary Table 19: Hexagonal fraction – one-way ANOVA for Supplementary Fig. 6b (ϕ_{SIL} = 0. 52, light grey bars)

ANOVA table	SS	DF	MS	F (DFn, DFd)	P value
Treatment (between columns)	0.04258	2	0.02129	F (2, 12) = 4.097	P = 0.0440
Residual (within columns)	0.06235	12	0.005196		
Total	0.1049	14			

Tukey's multiple comparisons test	Mean Diff.	95% CI of diff.	Significant?	Summary
35.4° vs. 36.3°	0.1180	-0.006102 to 0.2422	No	ns
35.4° vs. 37.4°	0.01845	-0.1106 to 0.1475	No	ns
36.3° vs. 37.4°	-0.09958	-0.2160 to 0.01687	No	ns

Supplementary Table 20: Comparison of contact angle measurement for small increases in ϕ_{SIL} (Supplementary Fig. 5a)

Unpaired t test with Welch's correction	
P value	0.0057
P value summary	**
Significantly different? (P < 0.05)	Yes
One- or two-tailed P value?	Two-tailed
Welch-corrected t, df	t=3.397 df=11.30
How big is the difference?	
Mean ± SEM of column A	27.03 ± 0.1722 N=7
Mean ± SEM of column B	27.99 ± 0.2221 N=7
Difference between means	0.9549 ± 0.2811
95% confidence interval	0.3382 to 1.571
R squared	0.5053
F test to compare variances	
F,DFn, Dfd	1.663, 6, 6
P value	0.5522
P value summary	ns
Significantly different? (P < 0.05)	No

Supplementary Table 21: Packing fractions for Fig. 2n-r and Supplementary Fig. 3e-h

			**Hexagonal**		**Square**		**Amorphous**		**No-Pack**	
ϕ_{SIL}	θ_{DIB}	*n*	average	st. dev.	average	st. dev.	average	st. dev.	average	st. dev.
**0.35**	9.9	3	0.28	0.08	0.08	0.03	0.10	0.02	0.53	0.08
**0.50**	25.8	5	0.37	0.03	0.17	0.05	0.18	0.04	0.26	0.07
**0.57**	33.2	5	0.36	0.06	0.17	0.05	0.10	0.02	0.34	0.03
**0.59**	35.3	6	0.43	0.06	0.16	0.06	0.14	0.04	0.25	0.06
**0.60**	36.3	5	0.31	0.05	0.17	0.04	0.16	0.04	0.33	0.07
**0.65**	41.6	5	0.28	0.04	0.16	0.02	0.22	0.05	0.31	0.06
**0.80**	57.5	6	0.17	0.06	0.12	0.04	0.40	0.05	0.27	0.11

Supplementary Table 22: Packing fractions for Fig. 3c and Supplementary Fig. 6

			Hexagonal		Square		Amorphous		No-Pack	
ϕ_{SIL}	x_{POPC}	n	average	st. dev.	average	st. dev.	average	st. dev.	average	st. dev.
**0.59**	0.00	6	0.43	0.06	0.16	0.06	0.14	0.04	0.25	0.06
**0.60**	0.00	5	0.31	0.05	0.17	0.04	0.16	0.04	0.33	0.07
**0.61**	0.00	4	0.28	0.05	0.15	0.07	0.19	0.03	0.36	0.03
**0.55**	0.11	3	0.47	0.01	0.14	0.04	0.17	0.03	0.20	0.02
**0.55**	0.13	5	0.50	0.07	0.11	0.03	0.12	0.04	0.25	0.07
**0.55**	0.16	5	0.30	0.06	0.16	0.05	0.14	0.04	0.38	0.07
**0.52**	0.18	4	0.40	0.04	0.10	0.03	0.15	0.02	0.33	0.03
**0.52**	0.20	6	0.28	0.08	0.12	0.03	0.17	0.02	0.40	0.09
**0.52**	0.23	5	0.38	0.08	0.14	0.03	0.14	0.04	0.32	0.05

Supplementary Table 23: Packing fractions for Fig. 3d

				Hexagonal		Square		Amorphous		No-Pack	
ϕ_{SIL}	x_{POPC}	f_D	n	average	st. dev.	average	st. dev.	average	st. dev.	average	st. dev.
**0.60**	0.00	2.00	4	0.28	0.05	0.15	0.03	0.19	0.04	0.36	0.03
**0.60**	0.00	0.50	5	0.31	0.05	0.17	0.04	0.16	0.04	0.33	0.07
**0.60**	0.00	0.25	3	0.33	0.08	0.11	0.04	0.17	0.05	0.37	0.02
**0.55**	0.13	2.00	3	0.22	0.03	0.08	0.01	0.28	0.06	0.40	0.09
**0.55**	0.13	0.50	5	0.50	0.07	0.11	0.03	0.12	0.04	0.25	0.07
**0.55**	0.13	0.25	4	0.17	0.05	0.11	0.02	0.26	0.09	0.43	0.14
**0.52**	0.20	2.00	3	0.29	0.06	0.16	0.02	0.25	0.04	0.27	0.02
**0.52**	0.20	0.50	6	0.28	0.08	0.12	0.03	0.17	0.02	0.40	0.09
**0.52**	0.20	0.25	5	0.16	0.04	0.16	0.04	0.21	0.04	0.43	0.09

Supplementary Table 24: Contact angle measurement for Fig. 1f-g

			θ_{DIB}	
ϕ_{SIL}	x_{POPC}	n	average	st. dev.
**0.20**	0.00	3	6.03	0.75
**0.35**	0.00	4	16.32	0.34
**0.50**	0.00	6	26.52	1.68
**0.57**	0.00	3	34.61	0.93
**0.65**	0.00	6	41.86	1.34
**0.80**	0.00	4	58.56	1.81
**0.65**	0.20	6	48.42	0.46
**0.65**	0.25	4	49.57	0.94
**0.65**	0.33	5	53.77	3.76
**0.65**	0.50	5	61.42	3.17
**0.65**	0.66	4	68.17	0.99
**0.65**	0.75	3	73.54	0.59
**0.65**	0.80	4	78.82	0.98
**0.65**	1.00	1	85.25	3.88

Supplementary Table 25: Linear regression for aqueous phase used in electrophysiological recordings (Supplementary Fig. 5c)

**Best-fit values**	
Slope	88.27 ± 4.722
Y-intercept when X=0.0	-2.602 ± 2.269
X-intercept when Y=0.0	0.02948
1/slope	0.01133
**95% Confidence Intervals**	
Slope	77.75 to 98.79
Y-intercept when X=0.0	-7.657 to 2.453
X-intercept when Y=0.0	-0.03150 to 0.07762
**Goodness of Fit**	
R square	0.9722
Sy.x	1.181
**Is slope significantly non-zero?**	
F	349.4
DFn, DFd	1.000, 10.00
P value	< 0.0001
**Deviation from zero?**	Significant
**Data**	
Number of X values	3
Maximum number of Y replicates	5
Total number of values	12
Number of missing values	3
**Equation**	Y = 88.27*X - 2.602

Supplementary Notes

Supplementary Note 1: Formation of droplet interface bilayers

In a DIB, the surface tension of the bilayer (γ_b) is related to the surface tension of the two monolayers (γ_m) through:

$$\gamma_b = 2\gamma_m + \Delta F \quad (1)$$

ΔF is the work required to form the bilayer per unit area. ΔF can be described as the free energy of adhesion per unit area. Bilayer formation occurs spontaneously between the two droplets when $\Delta F < 0$. γ_b and ΔF can be calculated from the equilibrium contact angle (θ_{DIB}) (Fig. 1a) of the adhering droplets and γ_m as described by Young from (Supplementary Fig.1b):

$$\gamma_b = 2\gamma_m cos\ \theta \quad (2)$$

By substituting Supplementary Eq. 2 into Supplementary Eq. 1, we arrive at the Young and Dupree relationship[1]:

$$-\Delta F = 2\gamma_m(1 - cos\ \theta) \quad (3)$$

From previous reports, $\gamma_m \approx 1$ mN m^{-1} in an excess of phospholipids[2]. From our measurements for DPhPC-only lipids, γ_m was measured between 2.0-3.5 mN m^{-1} depending on the oil composition (Supplementary Fig. 1d). We observed that an increase in θ_{DIB} (dependent on ϕ_{SIL}) was a direct reflection of an increase in ΔF (Supplementary Fig. 1f). However, we were interested in how the geometrical constraint of θ_{DIB} changed the packing of droplets in 3D space. From this, experiments were performed to investigate how ϕ_{SIL}, lipid concentration (0.05–4 mM) and composition, droplet volume (0.52–200 nL), and system temperature (4–60°C) changed θ_{DIB} in pairs of 75 nL PBS droplets.

We found that θ_{DIB} rapidly decreased with decreasing concentration of lipid below a critical DPhPC concentration of 0.91 mM (calculated from the exponential fit, $R^2 = 0.97$, see Supplementary Table 4) at $\phi_{SIL} = 0.65$ (Supplementary Fig. 1g).

However, no significant change was found for lipid concentrations above 1 mM. Stable DIBs could not be formed below 0.1 mM DPhPC (at a concentration of 0.05 mM DPhPC, $n = 6$ DIBs ruptured and coalesced upon initial formation). We assumed that the concentration dependence of the lipid on θ_{DIB} (up to a critical concentration of 1 mM) was due to the loose packing of the lipid monolayer below the critical DPhPC concentration[3]. However, to confirm this, further investigation is required.

The lipid and oil compositions greatly changed θ_{DIB}. The increase in θ_{DIB} with respect to ϕ_{SIL} (Fig. 1f) reflects the decreased solubility of lipid in the oil solution, which has been reported previously[4]. Moreover, different lipid compositions at a fixed concentration (1 mM) and fixed oil composition (0.65 ϕ_{SIL}) demonstrated different θ_{DIB} values (Fig. 1g, Supplementary Fig. 1h). These changes reflected the distinct solubilities of different lipids in the oil phase. It has previously been reported that a decrease in alkyl tail length and unsaturation of PC lipids decreases the solubility of the lipid in silicone oil[5]. We observed the same when moving from DPhPC to DMPC, to the extent that DIBs could not be formed from DMPC because of precipitation of the lipid in the oil. We acknowledge that if we had used DMPC above its melting transition temperature (T_m = 24°C), we would have been able to form stable DIBs[6]. Modifying the temperature (4-60°C) (Supplementary Fig. 1i) or droplet volume (50-200 nL) (Supplementary Fig. 1j) did not significantly change θ_{DIB} (see Supplementary Tables 3 and 5). We attribute the θ_{DIB} independence with respect to temperature and droplet volume to the lack of a T_m for DPhPC[7] and an insignificant change in the Laplace pressure in sub-nL to nL droplets, respectively.

Supplementary Note 2: 3D printing of droplet networks

In our printing setup, we eject aqueous droplets into an oil solution containing lipids. Typically, DPhPC is dissolved at 1 mM in an oil mixture of undecane and silicone oil at $\phi_{SIL} = 0.65$[8]. Our lipid choice is based on the constant fluidity of DPhPC membranes (a lack of apparent gelling temperature[7]), which has been shown to be ideal for studying pore-forming toxins and certain membrane proteins at the single-molecule level in black lipid membranes and DIBs[9]. Our choice of oil is based on controlling the viscosity and density of the printing medium as well as decreasing the solubility of the lipid in the solution for DIB formation[8]. We used PBS as the fixed aqueous phase for

our experiments. Each droplet was generated on demand via a piezoelectric droplet generator – typically at a printing frequency of 0.5 s^{-1} – for automatic ejection of picolitre-sized aqueous droplets in a lipid/oil solution.

During setup, droplet size is controlled by tuning the pulse amplitude and duration of the piezo-electric actuator. Once droplet sizes were calibrated to approximately 100 μm in diameter, the stepping distance between droplets during the automatic print was set to an appropriate value to ensure contact between subsequent droplets. This spacing was usually 85–95 μm depending on the droplet size. Importantly, droplet networks were patterned following a hexagonal close-packing (hcp) arrangement.

Supplementary Note 3: Theoretical packing constraints and observed packing in 3D-printed droplet networks

Geometrical packing constraints of droplet networks

The geometrical packing constraints are based on work investigated by Princen *et al*[10]. Similar to DIBs, oil-in-water emulsions stabilised by surfactants adhere and form a finite contact angle at the interface under certain conditions (paraffin oil droplets in an aqueous phase comprising 0.25 % sodium laurate, 0.25 % sodium oleate, and 0.6 M NaCl)[11]. Akin to DIB formation, the observed contact angles between droplets are a result of lowering surface tension in the system. Princen *et al* calculated the contact angles required to macroscopically exclude the continuous water phase between clusters of three to four adhesive oil-in-water droplets (stabilised by a surfactant). Here, we prove trigonometrically and experimentally (by varying θ_{DIB} with ϕ_{SIL}), the θ_{DIB} required for excluding the continuous oil phase at the centre of three and four droplets arranged where their centre-to-centre distances lie at the corners of an equilateral triangle and a regular tetrahedron, respectively.

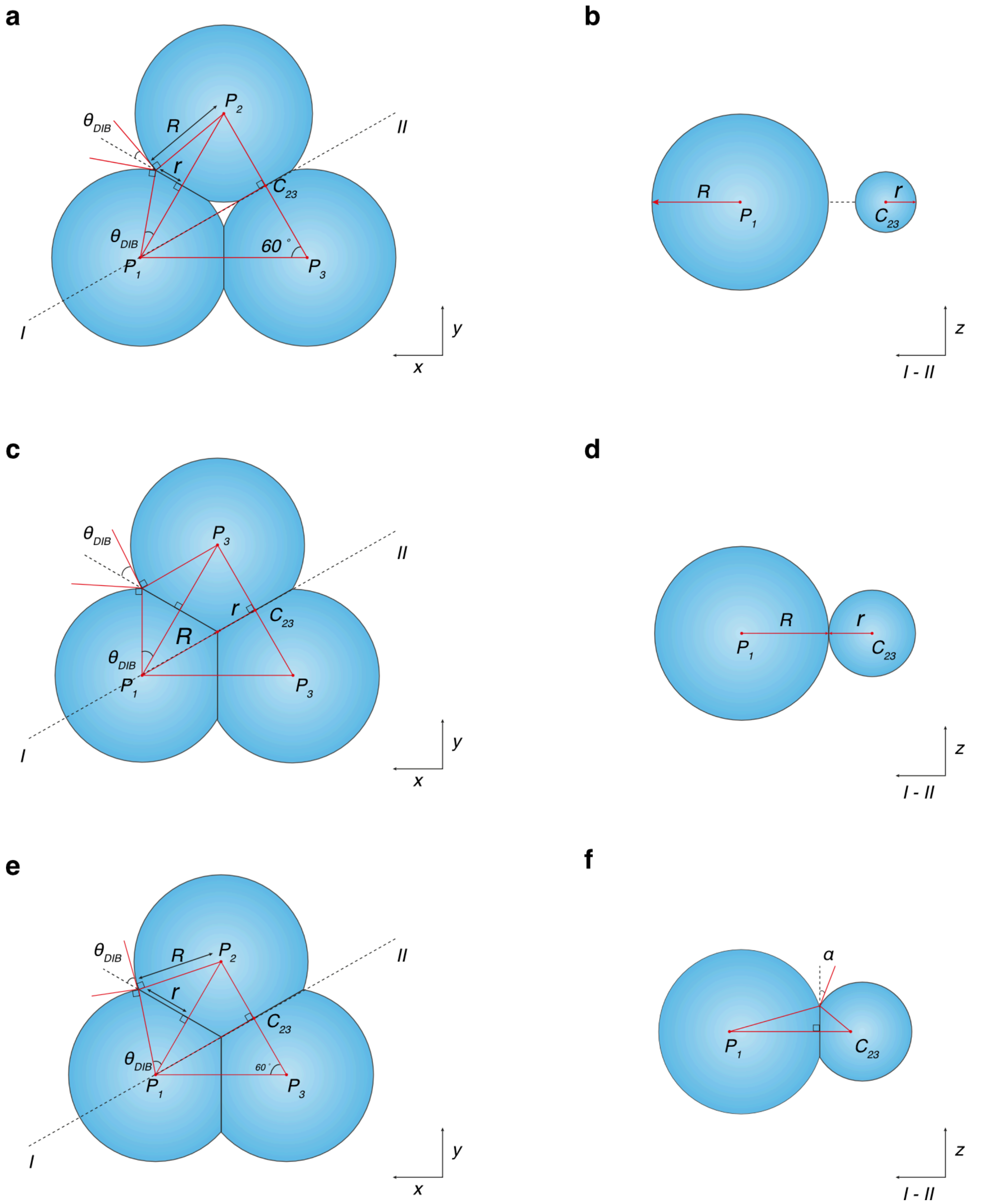

Supplementary Notes Fig. 1. a-f, Triplets of droplets of equal sizes arranged where their centre-to-centre distances ($P_1P_2,\ P_2P_3,\ P_2P_3$) lie at the corners of an equilateral tringle. R is the radius of the spherical cap and r is the radius of the bilayer formed between two droplets. In **a-b**, $\theta_{DIB} < 30°$; in **c-d,** $\theta_{DIB} = 30°$; and in **e-f**, $\theta_{DIB} > 30°$

***Three droplets**:* When three droplets of equal size form a 2D cluster, the minimum free energy of the system is obtained when the centre-to-centre distances are arranged at corners of an equilateral triangle (Supplementary Notes Fig. 1). Each

droplet adopts the shape of a doubly truncated sphere with a radius of R. The radius of the bilayer between each droplet is defined as r. As R is the same for each droplet, the centre-to-centre distances and r can be calculated from:

$$P_1P_2 = P_2P_3 = P_2P_3 = 2Rcos\theta_{DIB} \tag{4}$$

$$r = Rsin\theta_{DIB} \tag{5}$$

The centre-to-bilayer-centre distance (P_1C_{23}) (Supplementary Notes Fig. 1) can be calculated from:

$$P_1C_{23} = 2Rcos\theta_{DIB}sin60^{\circ} \tag{6}$$

As $sin60^{\circ} = \frac{\sqrt{3}}{2}$, this simplifies to:

$$P_1C_{23} = \sqrt{3}Rcos\theta_{DIB} \tag{7}$$

The three central monolayer edges must touch to exclude, macroscopically, the central continuous phase (Supplementary Notes Fig. 1c-d). For this to happen:

$$P_1C_{23} = R + r \tag{8}$$

As $P_1C_{23} = R\sqrt{3}cos\theta_{DIB}$ and $r = Rsin\theta_{DIB}$, substituting into Supplementary Eq. 8:

$$R\sqrt{3}cos\theta_{DIB} = R + Rsin\theta_{DIB} \tag{9}$$

$$\sqrt{3}cos\theta_{DIB} = 1 + sin\theta_{DIB} \tag{10}$$

$$\sqrt{3} = \frac{1}{cos\theta_{DIB}} + \frac{sin\theta_{DIB}}{cos\theta_{DIB}} \tag{11}$$

$$\sqrt{3} = sec\theta_{DIB} + tan\theta_{DIB} \quad (12)$$

$$sec^2\theta_{DIB} = (\sqrt{3} + tan\theta_{DIB})^2 \quad (13)$$

As $sec^2\theta_{DIB} = 1 + tan^2\theta_{DIB}$ this simplifies to:

$$1 + tan^2\theta_{DIB} = 3 + tan^2\theta_{DIB} - 2\sqrt{3}tan\theta_{DIB} \quad (14)$$

$$2\sqrt{3}tan\theta_{DIB} = 2 \quad (15)$$

$$\theta_{DIB} = tan^{-1}\left(\frac{1}{\sqrt{3}}\right) \quad (16)$$

$$\theta_{DIB} = 30° \; (Q.E.D) \quad (17)$$

Therefore, when $\theta_{DIB} < 30°$, the monolayers at the centre will not touch and a continuous oil phase will be running through the middle of the assembly (Supplementary Notes Fig. 1a-b). Experimentally, this was observed for ϕ_{SIL} = 0.2 (θ_{DIB} = 6.0 ± 0.7°) and ϕ_{SIL} = 0.5 (θ_{DIB} = 26.5 ± 1.7°) (Supplementary Fig. 4a,b and j). When θ_{DIB} = 30°, the monolayers will touch at a point and, macroscopically, no continuous oil phase will be running through the centre (Supplementary Notes Fig. 1c-d). At $\theta_{DIB} > 30°$, this central point will collapse to form the trigonal border comprising of three monolayer vertices meeting at angles of 120°. The radius of this contact and the cross-section contact angle (α) increases with θ_{DIB} (Supplementary Notes Fig. 1e-f). Experimentally, this was observed for ϕ_{SIL} = 0.6 (θ_{DIB} = 36.6°, calculated from Fig. 1h), ϕ_{SIL} = 0.65 (θ_{DIB} = 41.9 ± 1.3°), and ϕ_{SIL} = 0.8 (θ_{DIB} = 53.4 ± 0.8°) (Supplementary Fig. 4c, d, e and k).

Four droplets: When four droplets of equal size form a 3D cluster, the minimum free energy is reached when the centre-to-centre distances are arranged at the corners of

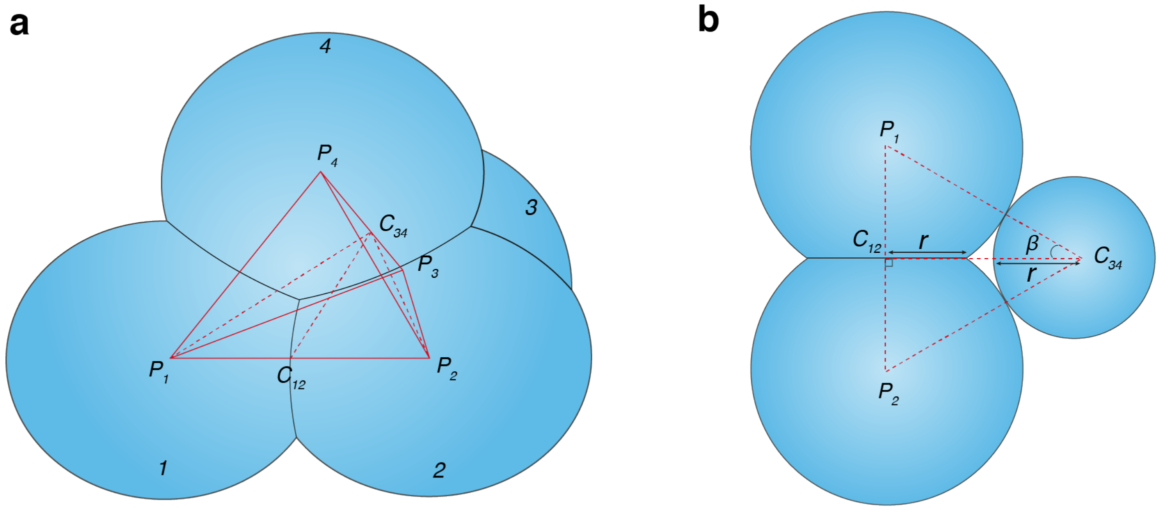

Supplementary Notes Fig. 2. a, A quartet of droplets of equal sizes arranged where their centre-to-centre distances lie at the corners of a regular tetrahedron. Droplet numbers are labelled on the diagram with the centre of each droplet indicated by P. **b**, The plane ($P_1P_2C_{34}$) (as indicated in **a**) of the centre-to-centre distances of droplets 1 and 2 (P_1P_2) to the centre of the bilayer formed between droplets 3 and 4 (C_{34}), drawing an isosceles triangle between their centres at $\theta_{DIB} < 35.3°$. The radii of the bilayers are indicated by r.

a regular tetrahedron (Supplementary Notes Fig. 2a). Each droplet adopts the shape of a truncated sphere with a radius of R. The required θ_{DIB} for the four monolayers to touch at the central tetrahedral pocket (θ_c) can be proven by considering the plane between the centre-to-centre distances of droplets 1 and 2 (P_1P_2) and the centre of the bilayer formed between droplets 3 and 4 (C_{34}). The plane is marked in Supplementary Notes Fig. 2a with a 2D projection shown in Supplementary Notes Fig. 2b. The radii of the bilayers (r) are marked in Supplementary Notes Fig. 2b. For the monolayers to touch at the tetrahedral centre:

$$C_{12}C_{34} = 2r \tag{18}$$

where C_{12} is the centre of the bilayer of droplets 1 and 2. We know from Supplementary Eq. 5 that $r = Rsin\theta_{DIB}$, and therefore:

$$C_{12}C_{34} = 2Rsin\theta_{DIB} \tag{19}$$

Supplementary Eq. 19 can also be written as:

$$C_{12}C_{14} = P_1C_{34}cos\beta \quad (20)$$

From Supplementary Eq. 7, $P_1C_{34} = P_2C_{34} = \sqrt{3}cos\theta_{DIB}$ and substituting into Supplementary Eq. 20 gives:

$$C_{12}C_{34} = R\sqrt{3}cos\theta_{DIB}cos\beta \quad (21)$$

where $cos\beta$ can be calculated from the law of cosines. As $P_1C_{34} = P_2C_{34}$, we define:

$$P_1{P_2}^2 = P_1{C_{34}}^2 + P_1{C_{34}}^2 - 2P_1{C_{34}}^2cos2\beta \quad (22)$$

$$P_1{P_2}^2 = 2P_1C_{34}(1 - cos2\beta) \quad (23)$$

As $(1 - cos2\beta) = 2sin^2\left(\frac{2\beta}{2}\right)$:

$$P_1{P_2}^2 = 4P_1{C_{34}}^2sin\beta \quad (24)$$

$$sin\beta = \frac{P_1P_2}{P_1C_{34}} \quad (25)$$

From Supplementary Eq. 4 and 7, $P_1P_2 = 2Rcos\theta_{DIB}$ and $P_1C_{34} = R\sqrt{3}cos\theta_{DIB}$, respectively. From this, Supplementary Eq. 25 simplifies to:

$$sin\beta = \frac{2Rcos\theta_{DIB}}{R\sqrt{3}cos\theta_{DIB}} \quad (26)$$

$$sin\beta = \frac{1}{\sqrt{3}} \quad (27)$$

$$cos\beta = \frac{\sqrt{2}}{\sqrt{3}} \quad (28)$$

Substituting Supplementary Eq. 20 and 28 into Supplementary Eq. 21:

$$2Rsin\theta_{DIB} = R\sqrt{3}cos\theta_{DIB}\frac{\sqrt{2}}{\sqrt{3}} \quad (29)$$

$$\frac{sin\theta_{DIB}}{cos\theta_{DIB}} = \frac{\sqrt{2}}{2} \quad (30)$$

$$\theta_{DIB} = tan^{-1}\frac{\sqrt{2}}{2} \quad (31)$$

$$\theta_{DIB} = \theta_c = 35.3° \; to \; 3 \; s.f. (Q.E.D) \quad (32)$$

Therefore, when $\theta_{DIB} < 35.3°$, the monolayers at the centre will not touch and there will be a continuous oil phase at the centre of the tetrahedral pocket. This was experimentally observed for $\phi_{SIL} = 0.5$ ($\theta_{DIB} = 26.5 \pm 1.7°$) (Supplementary Fig. 4f and l). Interestingly, at $\phi_{SIL} = 0.2$ ($\theta_{DIB} = 6.0 \pm 0.8°$), a tetrahedron could not be formed – the top droplet could not be supported by the bottom three and fell into the middle of the assembly. When $\theta_{DIB} = 35.3°$, the monolayers will touch at a point. At $\theta_{DIB} > 35.3°$, this point will collapse so the four monolayers meet at the tetrahedral centre at 109.3° (to 4 s.f.) angles, creating a tetrahedral border where macroscopically, there will be no continuous oil phase running through the centre. It was experimentally difficult to confirm the closing of the tetrahedral border: for $\theta_{DIB} > 35.3°$ at $\phi_{SIL} = 0.6$ ($\theta_{DIB} = 36.6°$, calculated from Fig. 1h), $\phi_{SIL} = 0.65$ ($\theta_{DIB} = 41.9 \pm 1.3°$), and $\phi_{SIL} = 0.8$ ($\theta_{DIB} = 53.4 \pm 0.8°$), repeated diffraction patterns were observed at the tetrahedral border (Supplementary Fig. 4g, h, i and m). However, we assumed the absence of a visible hole indicated the closing of the border.

The trigonal and tetrahedral borders close and thus exclude the continuous oil phase (on the micrometre scale) at $\theta_{DIB} < 30°$ and $\theta_{DIB} < 35.3°$, respectively. We define the critical angle (θ_c) of 35.3° as the minimum θ_{DIB} required to exclude the continuous

oil phase at the tetrahedral border enclosed by four droplets arranged where their centres are located at the corner of a regular tetrahedron. In the next section, $\theta_{DIB} \approx \theta_c$ will become apparent for maximising hexagonal close-packing (hcp) of droplets in 3D-printed droplet networks.

We note that as a result of curvature constrains on the monolayer, the continuous oil phase will be present between the trigonal and tetrahedral borders on the nanometre scale[12]. We have not proven this experimentally.

The equilibrium contact angle directs the packing structure of 3D-printed droplet networks

From our experiments, when $\theta_{DIB} \approx \theta_c$ (35.3°), hcp of droplets in 3D-printed droplet networks is maximised. In brief, we reason this because when $\theta_{DIB} \approx \theta_c$, the tetrahedral arrangements in the lattice are generated with minimal distortions. When droplets are positioned in a hcp arrangement, groups of four droplets are arranged with their centres are at the vertices of a regular tetrahedron, as seen in Supplementary Notes Fig. 2 and 3[12]. To maximise hcp, these tetrahedral arrangements of droplets must be maintained in the position imposed by the printing nozzle. The maintenance of these tetrahedral arrangements depends on the value of θ_{DIB} and the adhesive energy of droplets.

When two droplets form a DIB, they deform from their initial spherical shape and assume a truncated sphere geometry. As described in Supplementary Eq. 4, their centre-to-centre distance L depends on θ_{DIB}, and specifically it reduces as θ_{DIB} increases. Consequently, two droplets of equal radius R forming a DIB will move towards each other of a distance equal to (R – L), which increases with increasing θ_{DIB}. The area of droplet-droplet contact, a circle with radius r as described in Supplementary Notes Fig. 1, also depends on θ_{DIB}, and specifically increases with θ_{DIB} (Supplementary Eq. 5). This is also reflected in the increase in adhesive energy between droplets as θ_{DIB} increases, shown in Supplementary Fig. 1f.

In 3D-printed droplet networks, at $\theta_{DIB} < \theta_c$ (i.e. $\phi_{SIL} = 0.35$) (Fig. 2d and i), the high fraction of no-packing (0.53 ± 0.08 area fraction) (Fig. 2n) and droplet rolling from the upper layers (65 ± 18% droplet excess) (Supplementary Fig. 3j) were attributed to the low adhesive energy between the droplets, and between the droplets and the

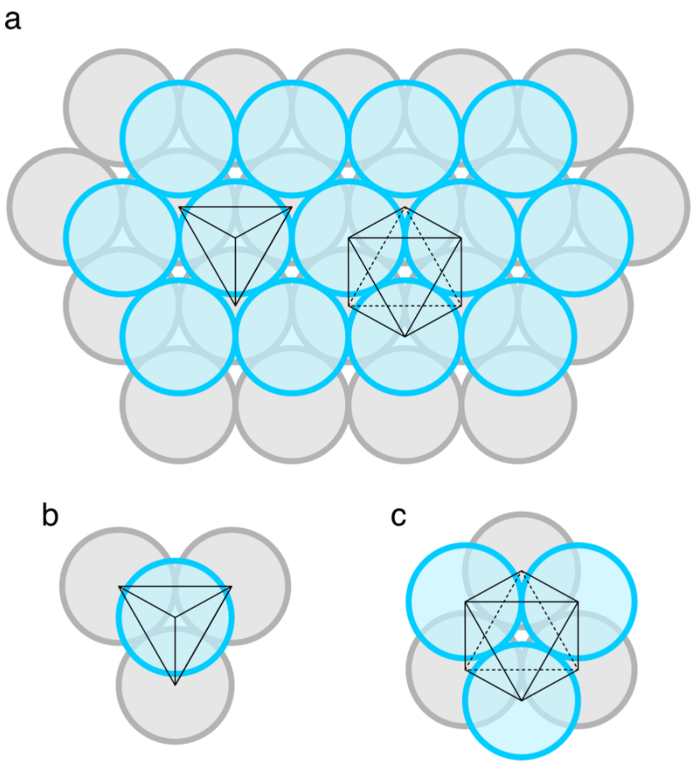

Supplementary Notes Fig. 3. a-c, Diagrams of spheres that are arranged in a hcp fashion (**a**) indicating the tetrahedral points (**b**) and octahedral points (**c**).

surface on which they were printed (Supplementary Fig. 1f and 7c). The observed high fraction of oil inclusions (0.18 ± 0.01 area fraction) (Supplementary Fig. 3i) was caused by the continuous oil phase present at the tetrahedral borders as θ_{DIB} (9.9°) << θ_c. Moreover, we also observed the highest variability in hexagonal packing (0.28 ± 0.08 area fraction) among printed networks. We attributed the high variability in packing structure to the relative movement of the droplets between each other before DIBs formed. Droplets fall at high speeds when ejected because of their low buoyancy in an oil composition where ϕ_{SIL} = 0.35, since the density of the oil (0.76 g mL^{-1}) is much lower than the aqueous solution (1.00 g mL^{-1}). This leads to a higher chance of relative movement of droplets from their desired printing location. Overall, the tetrahedral arrangements of groups of four droplets are not maintained because of low adhesive energy between droplets, leading to a packing structure that is mostly not-packed.

At θ_{DIB} >> θ_c (i.e. ϕ_{SIL} = 0.8) (Fig. 2h and m), the high fraction of amorphous packing (0.40 ± 0.05 area fraction) (Fig. 2r) and low fraction of oil inclusions (0.03 ± 0.01 area fraction) (Supplementary Fig. 3i) were attributed to the high adhesive energy between the droplets (Supplementary Fig. 1f), and between the droplets and the surface (Supplementary Fig. 7c) that distorted the packing structure to increase the

bilayer surface area. The high number of droplets falling from the upper layers (61 ± 24% droplet excess) (Supplementary Fig. 3j) was caused by the decrease in droplet centre-to-centre distances which created voids in the bottom layer of the networks and in which droplets from the upper layers could occupy. The high variability in droplet size (28 ± 8 coefficient of variation) (Supplementary Fig. 3k) also reflected how distorted the droplet shapes appeared within the structure. Overall, when $\theta_{DIB} >> \theta_c$ droplet centre-to-centre distances reduce, causing the droplets to move away from the initial position imposed by the printing nozzle. Tetrahedral arrangements are not maintained, and amorphous packing is observed predominantly in the networks.

When $\theta_{DIB} \approx \theta_c$ ($\phi_{SIL} = 0.59$, $\theta_{DIB} = 35.4°$) (Fig. 2f and k), we found the highest amount of hexagonal packing (0.43 ± 0.06 area fraction) (Fig. 2p), together with the lowest amount of oil inclusions (0.06 ± 0.01 area fraction) (Supplementary Fig. 3i), droplet rolling from the upper layers (13 ± 6% excess droplets) (Supplementary Fig. 3j), and droplet size variation (8.2 ± 1.5 coefficient of variation) (Supplementary Fig. 3k). We reason that when $\theta_{DIB} \approx \theta_c$, hcp is maximised in 3D-printed droplet networks because the adhesive energy between droplets is sufficient to allow the formation of stable structures, and there is minimal movement of droplets from their initial position (imposed by the printing nozzle) and their final position (reached once θ_{DIB} is reached) during printing. The minimal contraction in droplet centre-to-centre distance follows from the fact that θ_c corresponds to the minimum contact angle that allows exclusion of the continuous oil phase at the tetrahedral border in clusters of four droplets arranged as a regular tetrahedron, as discussed above. Moreover, $2\theta_c$ is the dihedral angle of a regular tetrahedron, therefore droplets are geometrically constrained as tetrahedrons when printed as tetrahedrons in a hcp arrangement. Overall, we find hcp packing is maximised in 3D-printed droplet networks at $\theta_{DIB} \approx \theta_c$ because the tetrahedral arrangements of droplets are maintained in the position imposed by the printing nozzle.

We note that in hcp packing, octahedral points are present (as indicated in Supplementary Notes Fig. 3c). At $\theta_{DIB} \approx \theta_c$, the continuous oil phase will be present at these octahedral points (which would close at $\theta_{DIB} = 45°$)[12]. However, we found this is not a problem for maximising hcp of droplets in 3D-printed droplet networks.

The bilayer composition changes the packing structure of 3D-printed droplet networks

We investigated if the composition of DIBs – at equal θ_{DIB} – changed the packing structure of our 3D-printed droplet networks. Therefore, we printed networks at θ_{DIB} values of 35.4°, 36.3°, and 37.2° (calculated from Eq. 1, main text) at various values of ϕ_{SIL} and x_{POPC}, to investigate how, at constant θ_{DIB}, the packing structure changed with different x_{POPC} (Supplementary Table 9).

At $x_{POPC} = 0$ (DPhPC only), hexagonal packing significantly dropped from a ϕ_{SIL} value of 0.59 to 0.60 (an equivalent change in θ_{DIB} of 35.4° to 36.3°) (Supplementary Fig. 6 group a). At a constant ϕ_{SIL} of 0.52 and varied x_{POPC} (0.18, 0.20, 0.22; equivalent to a θ_{DIB} of 35.5°, 36.3°, and 37.4°, respectively), we observed no significant difference in hexagonal packing (Supplementary Fig. 6 group c). At $\phi_{SIL} = 0.55$ and $x_{POPC} = 0.13$ ($\theta_{DIB} = 36.3°$), we observed the most hexagonal packing (0.50 ± 0.07 area fraction) in the networks (Supplementary Fig. 6 group b). In contrast to DPhPC-only lipid-oil solutions (Supplementary Fig. 6 group a), a higher θ_{DIB} significantly reduced hexagonal packing, specifically from 36.3° to 37.2° (Supplementary Fig. 6 group b). We confirmed that the changes in θ_{DIB} required for maximising hexagonal packing in the first layer at 0.00 x_{POPC} ($\theta_{DIB} = 35.4°$) and 0.13 x_{POPC} ($\theta_{DIB} = 36.3°$) were not because of changes in final θ_{DIB}, but changes in the kinetics of contact angle equilibration (Fig. 3).

The kinetics of contact angle equilibration maximises hexagonal packing

As discussed above, we found that $\theta_{DIB} \approx \theta_c$ is a key factor in controlling the amount of hcp in 3D-printed droplet networks. However, we also observed differences in the final packing structure of 3D-printed droplet networks at equal θ_{DIB}, but at different oil and lipid compositions (Supplementary Fig. 6).

We hypothesised that these differences in packing arose from differences in the kinetics of contact angle equilibration at different lipid/oil compositions. To prove this, we measured the non-equilibrium contact angle (θ) over time (Methods) in droplet pairs formed at different oil and lipid compositions leading to an equal $\theta_{DIB} = 36.3°$, calculated from Eq. 1 (main text) (Fig. 3b-d, cyan: $\phi_{SIL} = 0.60$ and $x_{POPC} = 0.00$; purple: $\phi_{SIL} = 0.55$ and $x_{POPC} = 0.13$; magenta: $\phi_{SIL} = 0.52$ and $x_{POPC} = 0.20$). The kinetics of

contact angle equilibration were different for DIBs formed with POPC compared to DIBs formed with just DPhPC – an initial fast phase was followed by a slow phase. For example, at $\phi_{SIL} = 0.55$ and $x_{POPC} = 0.13$ (purple line in Fig. 3b) a $\theta \approx 30°$ is reached within 2 seconds from contact of the droplets, while for $\phi_{SIL} = 0.60$ and $x_{POPC} = 0.00$ (cyan line in Fig. 3b) the same $\theta \approx 30°$ is reached after 6 seconds. However, despite an initial slower phase, θ_{DIB} was reached faster in the absence of POPC ($\phi_{SIL} = 0.60$ and $x_{POPC} = 0.00$) than when POPC is present in the lipid mixture. By comparing $\phi_{SIL} = 0.60$ and $x_{POPC} = 0.00$ (cyan line in Fig. 3b), and $\phi_{SIL} = 0.55$ and $x_{POPC} = 0.13$ (purple line in Fig. 3b), the former reaches θ_{DIB} within 500 s after contact of the droplet pair, while for the latter, θ_{DIB} is still not reached after 900 s (the final contact angle is reached within 1800 s). Notably, networks printed at conditions of $\phi_{SIL} = 0.55$ and $x_{POPC} = 0.13$ were significantly higher in hexagonal packing fraction than at conditions of $\phi_{SIL} = 0.60$ and $x_{POPC} = 0.00$ (Fig. 3c and Supplementary Table 15).

We hypothesised this significant increase in hexagonal packing from $\phi_{SIL} = 0.60$ and $x_{POPC} = 0.00$, to $\phi_{SIL} = 0.55$ and $x_{POPC} = 0.13$, was caused by the kinetics of contact equilibration in relation to the droplet ejection frequencies used when 3D printing. We typically used a printing frequency of 0.5 s^{-1} (as discussed in Methods and Supplementary Note 2) to form 3D-printed droplet networks, which corresponds to a time interval of 2 s between ejection of subsequent droplets (indicated as t_{drop} in Fig. 3b). At this printing frequency, each 7 x 8 droplet layer is completed in 225 s (t_{layer} in Fig. 3b) and a 7 x 8 x 4 (*x*, *y*, *z*) droplet network is completed after ≈ 900 s.

For droplet networks printed at a frequency of 0.5 s^{-1} and at $\phi_{SIL} = 0.55$ and $x_{POPC} = 0.13$ (purple line in Fig. 3b), by the time a new droplet was ejected, the contact angle at the previous droplet-droplet interface had reached a non-equilibrium value of 30° (t_{drop} in Fig. 3b). During printing of the whole network, a non-equilibrium contact angle between 30° and 35.3° is maintained in every DIB within the network for the entire formation of the printing process (approximately 900 s). In particular, by the time the first layer is formed (225 s, indicated as t_{layer} in Fig. 3b), each DIB forming in this layer has reached a non-equilibrium contact angle between 30° and 33°. We suggest that this maximises the formation of a regular 2D hexagonal packing in the first layer, from which 3D hcp can develop once every DIB in the network has reached equilibrium (approximately 1800 s). As discussed above, a contact angle of 30° correspond to the

minimum contact angle that excludes the continuous oil phase at the middle of a cluster of three droplets, arranged with their centres at the vertices of an equilateral triangle. Minimal centre-to-centre distance contractions and the geometrical constraint of $2\theta = 60°$ (the vertex angle of an equilateral triangle) maximises 2D hexagonal packing in the first printed layer.

We suggest that the combination of the printing frequency and the fast phase in the contact angle equilibration rate profile (Fig. 3b) allowed the first layer to hexagonally pack before the second layer was printed on top (Fig. 3b, t_{layer} = 225 s). The slow phase in the rate profile allowed the slow development of hcp from this 2D hexagonal packing throughout the network, as θ_{DIB} approximating θ_c was reached (after 900 s).

To prove that the significantly higher hexagonal packing fraction for $\phi_{SIL} = 0.55$ and $x_{POPC} = 0.13$ was caused by the optimal matching of the kinetics of contact angle equilibration and the printing frequency, we printed networks at a higher frequency, t_{fast}^{-1} = 2 s^{-1} (equal to a droplet ejection time interval of 0.5 s, indicated as t_{fast} in Fig. 3b and d), and at a lower frequency t_{slow}^{-1} = 0.25 s^{-1} (equal to a droplet ejection time interval of 4 s, indicated as t_{slow} in Fig. 3b and d). We observed that the hexagonal packing fraction at the original frequency t_{drop}^{-1} = 0.5 s^{-1} was significantly higher compared to networks printed at both a higher and lower frequency (Fig. 3d and Supplementary Table 16).

Overall, we confirmed our hypotheses that the regularity of the hexagonal lattice in the first layer was optimal when the conditions were such that $\theta_{DIB} \approx \theta_c$ in a droplet pair, and the printing frequency and the kinetics of contact angle equilibration were adjusted to maximise the initial formation of regular 2D hexagonal packing in the first layer, which could then develop into 3D hcp once subsequent layers were printed.

Surface composition and environmental humidity changed the packing of 3D-printed droplet networks

We observed changes in the regularity of hexagonal packing depending on the surface on which 3D droplet networks were printed. It has previously been reported that DIBs can be formed with glass[9]. We found that the contact angle with the surface ($\theta_{surface}$) (Methods and Supplementary Fig. 7a) decreased with quartz treated with a plasma cleaner, untreated quartz, and roughened glass (treated with 8M NaOH) (Supplementary Fig. 7b). Moreover, $\theta_{surface}$ was directly proportional to ϕ_{SIL} for plasma-treated quartz (Supplementary Fig. 7c). Importantly, θ_{DIB} was unaffected by $\theta_{surface}$ and demonstrated similar values (Supplementary Fig. 7d) of θ_{DIB} formed on PMMA. To form regular, hcp lattices in 3D-printed droplet networks, contact of the printed droplets with the surface was required, as descried in the main text (Supplementary Fig. 7g-h).

We found that an environmental humidity between 40–50% was optimal for generating hcp lattices in droplet networks. 3D printing below and above this threshold led to distortions in the networks and irregular packing structures (Supplementary Fig. 7i-j): below 40% humidity, droplets statically repelled each other when placed directly next to each other by the droplet-printer; above 50% humidity, droplets did not from an adhesive patch with the quartz surface, resulting in droplets rolling out of place due to viscous drag distortions by the nozzle.

Supplementary Note 4: Limiting factors for imaging droplet shapes within the 3D-printed droplet networks

The imaging of the 3D droplet geometry within the printed networks is challenging, especially in the layers away from the quartz surface, as observed in Fig. 2b and Supplementary Fig. 8c-g. Distortions of the apparent 3D droplet shape are caused by optical aberrations, which are due to the differences in refractive index of the aqueous (η = 1.33) and oil (η = 1.42) solutions. Moreover, 3D-printed droplet networks are a highly scattering media, which limits the imaging of the lipid bilayers in depth.

To further investigate optical aberrations in our system, we attempted to image a quartet of handmade droplets (75 nL, PBS) arranged as a regular tetrahedron, in which only the droplet at the top vertex contained a fluorophore (Supplementary Fig. 8h). In this simplified system, we observed the same aberration-artefacts in the top

droplet (Supplementary Fig. 8i-j) as those seen from the fluorescent droplets within the networks (Supplementary Fig. 8c-g). Specifically, the fluorescent signal was much weaker in the top droplet above the bilayers formed between the droplets in the bottom layer, particularly in the junction point between three droplets.

We concluded that these are spherical aberrations caused by curved oil/water interfaces and different light-path lengths, were due to the presence of oil pockets between the three bottom droplets and the glass. Further investigation is required to fully understand the optical properties of droplet networks.

Supplementary Methods

Imaging and reconstructing individual fluorescent droplets within 3D-printed networks. Droplet networks (10 x 12 x 4; *x*, *y*, *z*,) were 3D-printed following the map shown in Supplementary Fig. 8a. Buffer droplets and fluorescently labelled droplets (containing 10 μM Atto488) were patterned drop by drop and layer by layer by printing the two different aqueous solutions using two nozzles. The individual droplets were imaged by confocal microscopy using an Olympus Fluoview FV3000 inverted microscope with a UPlanSApo 20X/0.75 dry objective.

Measuring surface tensions. Interfacial energies of PBS in lipid (1 mM DPhPC) containing oil (ϕ_{SIL} = 0.2–0.8) were measured using a pendant drop tensiometer (IT Concept Tracker). A flat needle was used (0.52 mm in diameter). The density of the aqueous and oil phases was calculated by averaging in triplicate the weight of 10 mL volumes (accuracy of weighing balance was 0.1 mg).

Supplementary References

1. Schrader, M. E. Young-Dupre Revisited. *Langmuir* **11,** 3585–3589 (1995).
2. Dixit, S. S., Pincus, A., Guo, B. & Faris, G. W. Droplet Shape Analysis and Permeability Studies in Droplet Lipid Bilayers. *Langmuir* **28,** 7442–7451 (2012).
3. Venkatesan, G. A. *et al.* Adsorption Kinetics Dictate Monolayer Self-Assembly for Both Lipid-In and Lipid-Out Approaches to Droplet Interface Bilayer Formation. *Langmuir* **31,** 12883–12893 (2015).
4. Thiam, A. R., Bremond, N. & Bibette, J. From stability to permeability of adhesive emulsion bilayers. *Langmuir* **28,** 6291–6298 (2012).
5. Yanagisawa, M., Yoshida, T.-A., Furuta, M., Nakata, S. & Tokita, M. Adhesive force between paired microdroplets coated with lipid monolayers. *Soft Matter* **9,** 5891–7 (2013).
6. Taylor, G. J. & Sarles, S. A. Heating-Enabled Formation of Droplet Interface Bilayers Using Escherichia coli Total Lipid Extract. *Langmuir* **31,** 325–337 (2015).
7. Lindsey, H., Petersen, N. O. & Chan, S. I. Physicochemical characterization of 1,2-diphytanoyl-sn-glycero-3-phosphocholine in model membrane systems. *Biochim. Biophys. Acta* **555,** 147–167 (1979).
8. Graham, A. D. *et al.* High-resolution patterned cellular constructs by droplet-based 3D printing. *Sci. Rep.* **7,** 7004 (2017).
9. Bayley, H. *et al.* Droplet interface bilayers. *Mol. BioSyst.* **4,** 1191–19 (2008).
10. Princen, H. M. Geometry of clusters of strongly coagulated fluid drops and the occurrence of collapsed Plateau borders. *Colloids Surf. A* **9,** 47–66 (1984).
11. Aronson, M. P. & Princen, H. M. Contact angles associated with thin liquid films in emulsions. *Nature* **286,** 370–372 (1980).
12. Princen, H. M., Aronson, M. P. & Moser, J. C. Highly concentrated emulsions. II. Real systems. The effect of film thickness and contact angle on the volume fraction in creamed emulsions. *J. Colloid Interface Sci.* **75,** 246–270 (1980).
